# Bionano Interface Optimization for Rational Lateral Flow Assay Development

**DOI:** 10.1021/acsnano.6c04136

**Published:** 2026-05-01

**Authors:** Christy J. Sadler, Maya Miller, Kevion K. Darmawan, Jan P. Sandler, Ho-Cheung Ng, André Shamsabadi, Adam Creamer, Carol V. Robinson, Irene Yarovsky, Molly M. Stevens

**Affiliations:** † Department of Physiology, Anatomy and Genetics, Department of Engineering Science, 6396 University of Oxford, Oxford OX1 3QU, United Kingdom; ‡ Kavli Institute for Nanoscience Discovery, 6396 University of Oxford, Oxford OX1 3QU, United Kingdom; § Department of Materials, Department of Bioengineering, Institute of Biomedical Engineering, 4615 Imperial College London, London SW7 2AZ, United Kingdom; ∥ School of Engineering, 5376 RMIT University, Melbourne, Victoria 3001, Australia; ⊥ Research Complex at Harwell, Harwell Science and Innovation Campus, Didcot Oxfordshire OX11 0FA, United Kingdom; # Department of Chemistry, 6396 University of Oxford, Oxford OX1 3TA, United Kingdom

**Keywords:** lateral flow assay, protein corona, nanoparticle, proteomics, diagnostics

## Abstract

Point-of-care diagnostic tools, such as lateral flow assays (LFAs), play a critical role in disease management and outbreak control. LFAs detect the presence of target antigens in disease-relevant biofluids, utilizing nanoparticles (termed detection probes) to produce colorimetric readouts. However, significant intra- and interpatient variation in the biochemical composition of biofluids has downstream consequences for assay performance. Robust LFAs must be able to function alongside such variability to produce reliable and reproducible test outcomes. Beyond this, biofluids (such as serum) contain significant amounts of proteins, which can interact with detection probes used in LFAs to form a protein corona. The consequences of protein corona formation on LFA performance are poorly understood. Using a model antigen-biofluid LFA (human epidermal growth factor receptor 2 (HER2) and human serum), we observed significant discrepancies in LFA performance when using conventional nanoparticle functionalization methods, including the use of generic, nonhuman protein blocking agents. To overcome these performance differences, we developed a methodology for Bionano interface Optimization for LFA Design (termed BOLD). The BOLD workflow employs mass spectrometry-based proteomics to characterize the native protein corona, followed by formation of an engineered corona to produce an optimized bionano interface. We identified a specific protein (kininogen-1, KNG1) that demonstrated negative interference, significantly reducing the observed LFA test line intensity. This experimental finding is complemented by Molecular Dynamics simulations, which probe the binding modes of KNG1 to platinum nanoparticles. Further, through the employment of an apolipoprotein engineered corona (apolipoprotein A1, B, and C3), a robust LFA was developed, increasing test line intensity and significantly reducing intersample variation (with over a 4-fold improvement in the coefficient of variation). Overall, the BOLD workflow presents a method for the rational optimization of detection probes in LFAs through the characterization of the bionano interface to produce robust LFAs.

## Graphical abstract

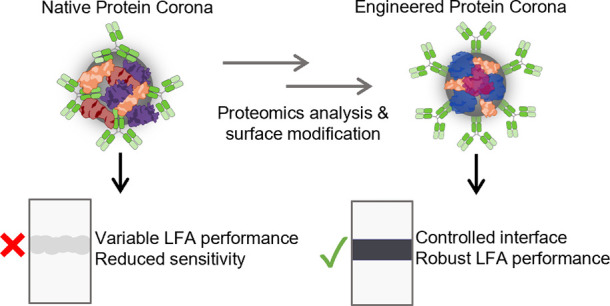

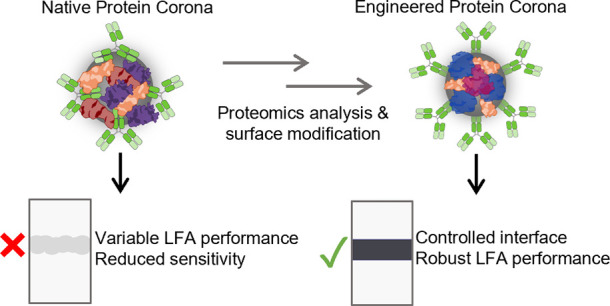

Point-of-care diagnostic tests, such as lateral flow assays (LFAs), have played a critical role in the successful management of disease. This has been apparent as part of the outbreak response measures during the COVID-19 pandemic, where LFAs were employed to aid implementation of public health measures and nonpharmaceutical interventions to curb disease transmission.^1–3^ LFAs are paper-based diagnostic tests that satisfy many of the ‘REASSURED’ criteria,^4,5^ particularly their low cost, rapidity, and ease of use. In LFAs, affinity agents (typically antibodies) are functionalized to nanoparticles (forming detection probes), which are used to produce a colorimetric signal at the test line. Commonly, colloidal gold nanoparticles (AuNPs) are used, producing a distinctive red signal at the test line. AuNPs are utilized due to their ease of functionalization (via adsorption methods) and suitability for large-scale synthesis.^6^ However, several methods have been utilized to produce signal amplification, including nanozymes,^7–11^ magnetic nanoparticles,^12–14^ and high-contrast optical nanoparticles (such as selenium nanoparticles^15,16^ or platinum nanoparticles, used without catalytic amplification^8,17^ ). Typically, LFAs produce qualitative test outcomes, where the presence or absence of a band at the test zone indicates disease status. However, quantification of disease biomarker abundance is becoming increasingly important.^6,18^ One specific example is the longitudinal monitoring of viral load in response to treatment regimens.^19^ Through the quantification of the test line intensity and correlation to biomarker concentration, treatment regimens can be altered to ensure viral load remains under a specified threshold, minimizing forward transmission of disease.

A range of human biofluids have been used in LFAs, including saliva,^20,21^ stool,^22,23^ and blood-based samples (including whole blood, serum, and plasma).^6,7^ Beyond this, LFAs have been developed to function with nonhuman biofluids, for example, the use of LFAs for the diagnosis of liver fluke in sheep and cattle.^24,24^ Depending on the design, the developed LFA can function with a range of biofluids, including C-reactive protein (CRP) LFAs, which can operate with either whole blood, serum, or plasma samples.^25,26^ Biofluids demonstrate significant intra- and interpatient variation, depending on clinical status and lifestyle factors,^27–29^ as well as sample handling.^30^ For human serum, variation can be observed in the biochemical composition (e.g., proteins, enzymes, lipids, small molecules),^29,31–33^ and physical characteristics (e.g., viscosity, pH).^34,35^ Developed LFAs must be able to function with heterogeneous samples (biochemical compositions and physical characteristics), with minimal variability in performance. To reduce assay variability, patient samples are commonly diluted to minimize the heterogeneity between samples,^36^ or exclusion criteria are applied (i.e., the test is not valid for use with samples with total cholesterol levels above 800 mg dL^–1^).^26^ It is well understood that interference can cause erroneous results in clinical biochemical assays, leading to both false positives and false negatives, depending on the nature of the interferent and the mechanism of interference.^37–41^ Interference can arise via a number of mechanisms, including chemical, spectral, physical, and enzymatic.^42^ Nonetheless, interference in LFAs is poorly understood and understudied, lacking clear documentation on the potential mechanisms of interference.^43,44^

Owing to the rapid nature of LFAs, visually detectable results are produced in a matter of minutes, with interactions in LFAs occurring on the order of seconds.^45,46^ This includes interactions between the target biomarker and biorecognition elements, as well as interactions between detection probes and components of the biofluid (such as proteins, lipoproteins, lipids, and metabolites). However, the interactions between detection probes and the biofluid sample are often overlooked in the development process. Upon exposure of detection probes to biofluids, a so-called protein corona can form, whereby components of the biofluid interact with and adsorb onto the nanoparticle surface to form a protein layer (i.e., protein corona). The protein corona forms due to nontargeted adsorption of proteins onto the nanoparticle surface, mediated by electrostatic, van der Waals, and hydrophobic interactions.^47–49^ Beyond the protein corona, it is understood that lipids can form adsorbed coronas around nanoparticles.^50^ The protein corona is a highly dynamic entity, initially driven by kinetic control, where highly abundant biofluid proteins (e.g., human serum albumin (HSA)) dominate the composition of the protein corona. Over time, the composition of the protein corona can shift, leading to the replacement of highly abundant biofluid proteins with higher-affinity proteins, as described by the Vroman effect.^51,52^ The fouling of proteins and lipids on nanoparticle surfaces can lead to several consequences for targeting nanoparticles.^50,53,54^ This includes loss of colloidal stability,^55,56^ exchange of biofluid proteins and detection antibodies,^57^ and masking of targeting ability.^58^ These consequences have primarily been studied for *in vivo* applications, where the formation of the protein corona can influence circulation efficiency.^57,59^ One area of interest is the engineering of the protein corona to exploit favorable interactions mediated by its presence. This includes the preformation of protein coronas to improve circulation efficiency and targeting ability.^60–62^ The consequences of protein and lipid corona formation have been less studied for in vitro diagnostic applications. Recent work by Rijal et al. investigated the preformation of a protein corona around AuNPs utilizing HSA and apolipoprotein A1 (APOA1), indicating that engineering the protein corona could modulate the observed LFA performance.^63^

In this work, we probe the interference effects of protein corona formation on half-dipstick LFA performance, using human epidermal growth factor receptor 2-biotin (HER2-biotin) as a model antigen. In the developed assay, we focus primarily on the interference effects between the biofluid, antigen, and platinum nanoparticle (PtNP) detection probe (used as a high-contrast optical detection probe, without catalytic signal amplification). Using serum from individual donors and traditional PtNP blocking agents (e.g., β-casein), we observed significant variation in LFA performance, and the presence of false-negative test results. To address the observed discrepancies, we developed a methodology that employs Bionano interface Optimisation for LFA Design (termed BOLD). The BOLD approach deviates from conventional optimization approaches, enabling rational selection of surface passivating proteins to engineer an idealized protein corona during nanoparticle functionalization, using three input parameters (nanoparticle, detection antibody, and sample matrix). Here, we showcase the BOLD method through characterization of the bionano interface and native protein corona using mass spectrometry-based proteomics. We identify several proteins, including highly abundant serum proteins (e.g., human serum albumin or apolipoproteins) and lower abundant serum proteins (e.g., fibronectin and kininogen-1), that are consistently identified in the native protein corona. To reduce the observed performance discrepancies, we optimize the bionano interface through the production of an engineered corona, using isolated human proteins to passivate the surface of PtNP detection probes. We identify kininogen-1 (KNG1) as a key interfering protein, which results in negative interference and near-complete loss of LFA signal when used as a surface passivating agent. Molecular Dynamics (MD) simulations are employed to characterize the adsorption of the surface binding region of KNG1 on PtNPs, including the investigation of amino acid residues involved in the binding mode and their behavior upon adsorption. Further, we develop an engineered apolipoprotein-based corona, which results in the production of a robust PtNP detection probe. The engineered corona PtNP detection probe is successful in standardizing the LFA performance, demonstrating a reduction in the LFA test line variability and significantly increasing the intensity of the test line. This includes the restoration of the LFA test line signal, where false-negative test results were previously observed. Overall, we demonstrate a BOLD methodology, which deviates from conventional LFA detection probe optimization methods by enabling rational optimization of the bionano interface to improve LFA robustness. The BOLD method can be readily translated to other LFA systems (varying the three input parameters: nanoparticle species, detection antibody, and biofluid sample), enabling the employment of engineered coronas for use with varying sample matrices and antigens.

## Results and Discussion

### LFA Robustness in Idealized Matrices

To assess the interference effects of human serum on LFA performance, we developed a model half-dipstick LFA to detect human epidermal growth factor receptor 2 (HER2) (Figure 1). HER2-biotin was utilized as a model serum-based antigen, alongside a polystreptavidin test line (capture probe). Pooled human serum samples were initially used to evaluate LFA performance (Table S1). The detection probe consisted of platinum nanoparticles (PtNPs) functionalized with trastuzumab (humanized mouse antibody against HER2) via physisorption. The PtNPs were initially characterized to assess their morphology, size, and surface charge. TEM micrographs of unfunctionalized PtNPs demonstrate a porous morphology, consistent with previously reported morphologies (Figure S1).^7–9,64^ The PtNPs demonstrated a surface charge of −52.4 ± 0.12 mV and a hydrodynamic diameter of 102.7 ± 1.49 nm (Table S2). PtNPs were utilized solely as high-contrast optical detection probes, without the employment of catalytic signal amplification. Initially, β-casein was used as a blocking agent to passivate the PtNP-trastuzumab surface, owing to its extensive use as a blocking agent in LFAs.^7,8,65^ It was assumed that, owing to the rapid association rate between biotin and streptavidin,^66^ the interaction between the biotinylated antigen and capture probe was constant in all sample matrices used. While this is an adequate first approximation, the presence of excessive free biotin (i.e., due to overconsumption of biotin supplements) has the potential to cause interference in immunoassays.^67,68^ However, supplementary biotin does not typically increase biotin concentration in the blood to a significant extent.^69^ Through the use of a pseudoconstant capture mechanism (i.e., biotin–streptavidin interactions), we were able to evaluate the interference effects that influenced detection probe performance.

**Figure 1:**
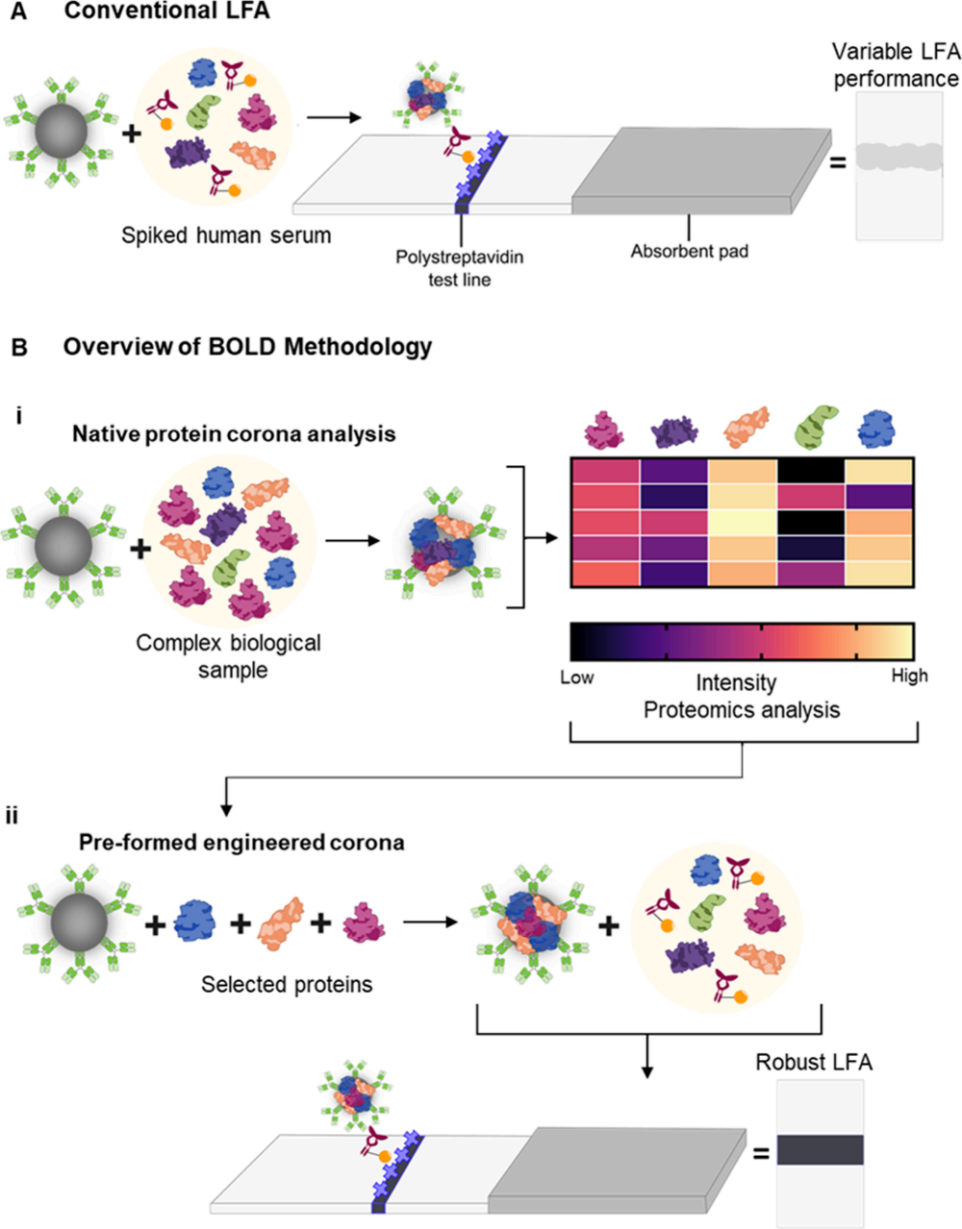
Schematic of the detection probes production following conventional vs Bionano interface Optimization for LFA Design (BOLD) methodologies, using HER2-biotin as a model antigen. (A) Conventional LFA, utilizing traditional nanoparticle functionalization methods, and the formation of a native protein corona. The LFA produces significant interpatient variation. (B) Bionano interface Optimization for LFA Design (BOLD) methodology to produce robust LFAs with minimal interpatient variation. (B­(i)) Formation of the native protein corona around PtNP detection probes on incubation with complex biological samples (i.e., human serum) from individual donors. The native protein corona is characterized using mass spectrometry-based proteomics. This requires three input parameters: nanoparticle species, detection antibody, and complex biological matrix. (B­(ii)) Formation of an engineered and rationally designed protein corona around PtNP detection probes, utilizing combinations of proteins identified from the proteomics analysis (i.e., apolipoproteins). The composition of the engineered protein corona is directly informed by the assessment of the composition of the native protein corona. The engineered corona formed around PtNP detection probes demonstrates improved performance in the model LFA, reducing variability in the generated test line intensity to produce a robust LFA.

Initially, we assessed the performance of the model LFA using idealized buffer (Dulbecco’s phosphate-buffered saline (DPBS) and DPBS with 0.05 v/v% Tween20 (DPBST)), spiked with HER2-biotin. Further, we employed two batches of pooled human serum (HS I and HS II), differing in biochemical composition (Table S1 and Figure S2). We assessed the signal (presence of antigen) and noise (absence of antigen) for each sample matrix. For each sample type, no test line intensity was measured in the absence of the antigen (i.e., no nonspecific binding) (Figure S3). Owing to the exogenous addition of the non-native antigen (i.e., biotinylated antigen), the test line intensities were expected to be comparable between sample matrices. However, significant differences in the generated test line intensity were observed between the four sample matrices used (Figure 2A,B). We observed that HS I produced a significantly weaker test line intensity compared to DPBS, DPBST, and HS II. This finding warranted further investigation into the origin of the observed visual discrepancies.

**Figure 2:**
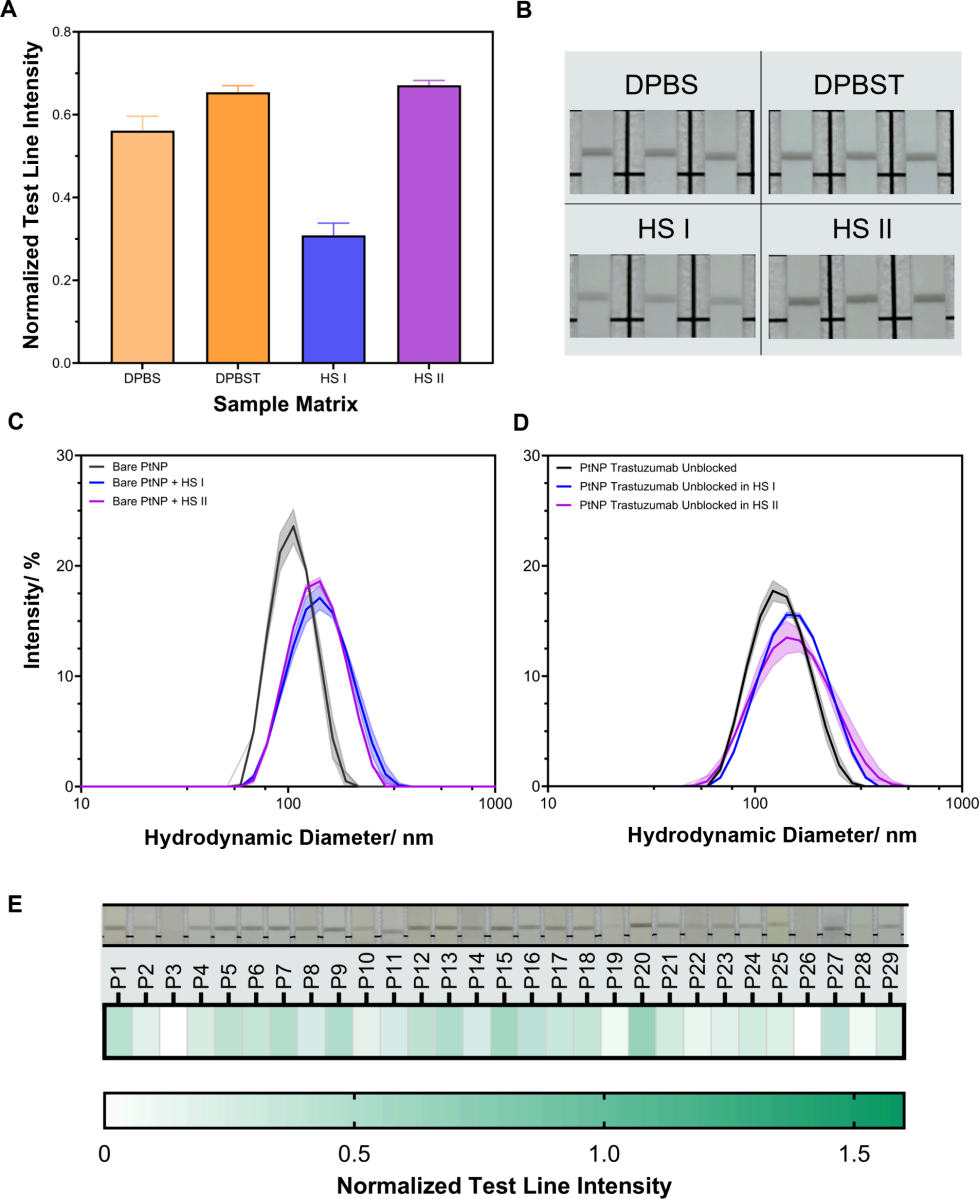
Assessment of performance variation in LFA using traditional generic protein (β-casein) surface passivation methods. (A, B) Assessment of LFA performance using four different spiked sample matrices- DPBS, DPBST (DPBS + 0.05 v/v% Tween20), HS I, and HS II. (A) Extracted LFA test line intensities using 500 pM of HER2-biotin spiked into the sample matrix, *n* = 3. (B) Photographs of the LFA test line using 500 pM of HER-biotin spiked into the sample matrix, *n* = 3. (C, D) Assessment of PtNP nanoparticle stability on incubation with HS I and HS II, determined by dynamic light scattering (DLS). (C) DLS of bare PtNPs incubated in HS I and HS II, measured after 15 min incubation with human serum. Data plotted as mean ± SD, *n* = 3. (D) DLS of the PtNP trastuzumab conjugate (without surface blocking), measured after 15 min incubation in HS I and HS II. Data plotted as the mean ± SD, *n* = 3. (E) Heatmap of the extracted LFA test line intensity for individual patient samples, with representative images depicted above. Normalized intensity represents the mean value (*N* = 29, *n* = 2).

### Stability of Platinum Nanoparticles in Human Serum

To probe the mechanism underpinning the LFA performance discrepancies, we assessed the stability of PtNPs in complex, protein-rich sample matrices. Briefly, interactions between PtNPs and human serum samples were probed using dynamic light scattering (DLS). Initially, bare (unfunctionalized) PtNPs were incubated in the two batches of pooled human serum (HS I and HS II) (Figure 2C). The samples were analyzed at four distinct time points (immediately after addition of HS, and after 15-, 30-, or 60 min incubation). The 15 min incubation times are most comparable to the interaction times in LFAs. DLS allowed for assessment of protein fouling and PtNP stability by monitoring changes in the hydrodynamic diameter and polydispersity. We observed an increase in the hydrodynamic diameter of bare PtNPs incubated in human serum (HS I and HS II), compared to bare PtNPs in DPBS (Figures 2C and S4). Moreover, the PtNPs incubated in HS I and HS II demonstrated a broadened size distribution, indicated by an increase in the PDI (Table S2). However, no significant aggregation of PtNPs was observed (PDI < 0.150), as indicated by a single size distribution. On extended incubation (30- and 60 min), no additional size increase or polydispersity was observed for both HS I and HS II (Figure S4 and Table S2). The increase in hydrodynamic radius and PDI is consistent with protein fouling through the adsorption of serum proteins onto the surface of PtNPs. Further, bare PtNPs were incubated in human serum for 15 min and subsequently washed three times (via centrifugation) to assess whether protein fouling persisted after sample purification. An increase in hydrodynamic diameter was observed compared to bare PtNPs (Table S3), with no significant aggregation observed (PDI < 0.150). This indicates that the adsorbed protein layer is retained after repeated centrifugation and washing steps.

The PtNP detection probes used in the aforementioned LFA were functionalized with targeting antibodies (trastuzumab) to enable specific antigen detection. Using DLS, we initially studied the stability of trastuzumab-conjugated PtNPs without blocking on incubation with HS I and HS II for 15 min (Figure 2D). Compared to the control PtNP trastuzumab conjugate (incubated in DPBS), an increase in hydrodynamic diameter was observed for both human serum samples. This is consistent with protein fouling on the PtNP trastuzumab conjugate surface (Figure 2D). On incubation in human serum, the PtNP conjugates demonstrated an appreciable broadening of the distribution of measured hydrodynamic diameters, indicated by an increased peak width and PDI (Table S4). This indicates an increase in polydispersity of the sample on incubation with human serum. The same trend was observed for PtNP-trastuzumab conjugate blocked with β-casein (as a surface passivating agent to reduce nonspecific binding), where size and polydispersity increases were observed (Figure S5). These findings confirm protein fouling on PtNP conjugate surfaces. The presence of protein fouling or protein corona formation may have downstream consequences on the observed performance of PtNP conjugates in LFAs.

### LFA Performance with Individual Human Serum Donors

To investigate the observed variability in LFA performance with pooled human serum samples (HS I and HS II), human serum samples from 29 healthy donors (Oxford Biobank) were utilized. The samples were selected based on total cholesterol levels (Table S5), owing to the known interference in other biochemical assays due to hypercholesterolemia.^39–41^ In healthy adult populations, cholesterol levels can vary significantly, and developed LFAs must be able to operate robustly under such variation. The samples were spiked with the HER2-biotin antigen and used in the aforementioned half-dipstick LFA.

For each of the 29 individual patient samples tested, no signal at the test line was observed on the addition of the sample without antigen spiking (i.e., no nonspecific binding) (Figure S6). On spiking the individual human serum samples with HER2-biotin, we observed significant variation in the test line intensity (Figures 2E and S7), demonstrated by a coefficient of variation (CV) of 0.58. Further, aggregation of PtNPs was observed at the base of the LFA strips, with the extent of aggregation varying between the human serum patient samples. This indicates loss of PtNP conjugate stability when performing the LFA. As with the pooled human serum, the origin of interpatient variation is hypothesized to originate from protein corona formation, leading to exchange of trastuzumab antibodies on the PtNP surface with serum proteins. This may result in heterogeneity of the functionalized PtNP population and variation in LFA performance. On spiking two individual human serum samples with HER2-biotin, no visible test line was distinguishable from the background (samples P3 and P26). The lack of apparent test line intensity would be interpreted as the absence of the HER2-biotin biomarker, indicating a false-negative test result. To assess the origin of the lack of observable test line intensity, DLS was employed to assess changes to hydrodynamic diameter and conjugate polydispersity. On incubation of PtNP conjugates with P3 or P26 samples, an increase in hydrodynamic diameter and PDI was observed (Table S6). The extent of hydrodynamic diameter increase is consistent with protein fouling, as observed for HS I and HS II (Table S4), whereby reduced test line intensities are associated with more significant increases to the conjugate hydrodynamic diameter.

While significant variation in test line intensity was observed across the 29 human serum samples, a correlation with total cholesterol was not found (Figure S8). This was indicated by variation in the observed test line intensity between samples containing comparable total cholesterol concentrations. Samples P3 and P26, that demonstrated no visible test line intensity, contained total cholesterol levels of 119.9 and 110.6 mg dL^–1^, respectively. Further, the test line intensities were plotted as a function of the ratio of total cholesterol to high-density lipoprotein (HDL). As with total cholesterol concentration, no correlation was observed. This suggests that varying cholesterol levels (total, high- and low-density lipoprotein) may not be the sole cause of assay interference in the model LFA. This finding warranted further investigation into the origin of the observed LFA interference.

### BOLD Methodology: Native Protein Corona Analysis

When performing the LFA using conventional PtNP functionalization and passivation methods (i.e., β-casein blocking agent), significant performance discrepancies were observed between patient samples. Due to the protein-rich nature of human serum, we hypothesized that a native protein corona could form around the PtNPs while running the LFA, which may have consequences on the resultant LFA performance.^43,63^ Recent work has characterized the native protein corona formation on AuNPs to probe LFA interference effects.^63^ However, the biochemical composition of the protein corona formed around core–shell PtNPs incubated in human serum has yet to be studied.^70^ Therefore, we develop a methodology to rationally select surface passivating agents through bionano interface optimization. The BOLD (bionano interface optimization for LFA design) method employed mass spectrometry-based proteomics (reverse phase-nano liquid chromatography/electrospray ionization-mass spectrometry (RP-nLC/ESI-MS)) to characterize the interface between the PtNP detection probes and individual human serum samples. This enabled identification of components of the native protein corona that can be used during PtNP functionalization to form an engineered corona. The BOLD methodology utilizes three system-specific input parameters (nanoparticle species, detection antibody, and complex sample matrix). We demonstrate the application of the BOLD methodology utilizing PtNPs functionalized with trastuzumab, and incubated in human serum. However, this methodology can be utilized with a broad range of nanoparticles, detection antibodies, and complex sample matrices.

We formed a native protein corona by incubating PtNP trastuzumab conjugates (omitting β-casein surface blocking) in human serum for 15 min under shaking (the maximum interaction time in LFAs). The samples were purified via centrifugation to remove excess, unbound proteins. After the final purification step, the protein concentration in the supernatant was analyzed using micro bicinchoninic acid (microBCA) assays (Figure S9 and Table S7) and UV–vis spectroscopy (Table S8). Analysis of the final supernatant demonstrated no significant unbound protein remaining in solution. This confirmed that downstream RP-nLC/ESI-MS analysis was probing proteins adsorbed onto PtNP surfaces, as opposed to unbound excess protein.

Initially, the proteomics sample preparation protocol (protein digestion and purification) was performed using pure protein samples (trastuzumab, HS I, and HS II), without the addition of PtNPs. This enabled the identification of proteins within the isolated samples before characterization of the native PtNP protein corona (Figure 3A,B and S10). Following this, the composition of the native protein corona formed on incubation of PtNP-trastuzumab conjugates with two pooled human serum samples (HS I and HS II) was assessed (Figure 3C). This enabled the development of a sample preparation utilizing PtNP conjugates, which enabled protein and peptide detachment from the PtNP surface. Label-free methods enabled assessment of the relative abundance of identified proteins, using normalized intensity to compare relative abundance both intra- and intersample. Absolute protein quantification was not employed in this workflow (i.e., through spiking of a control protein of known concentration), owing to variation between on-particle and in-solution digestion efficiency. On incubation with HS I and HS II, it was revealed that apolipoproteins (protein ID: APOA1, APOA2, APOB), fibronectin (protein ID: FN1), and immunoglobulin G (IgG, protein IDs: IGHG1, IGKC) demonstrated high intensity and relative abundance in the proteomics analysis (Figure 3C). This is in contrast to neat HS I and HS II, where human serum albumin (protein ID: HSA) was identified as the protein with the highest intensity and abundance (Figure 3A). As such, the protein corona formed around PtNP-trastuzumab conjugates was not restricted to highly abundant serum proteins alone.

**Figure 3:**
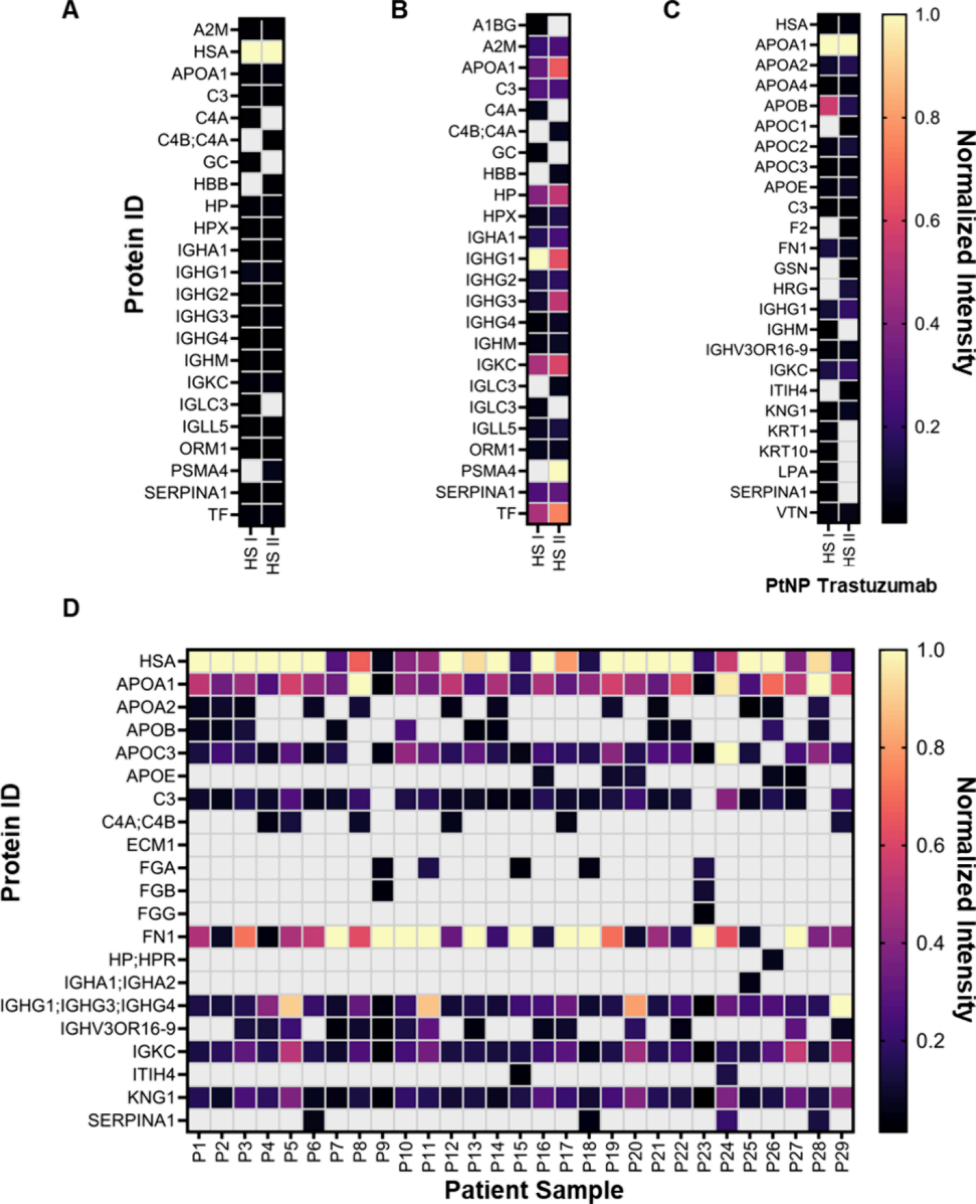
Mass spectrometry-based proteomics analysis of native protein coronas. (A) Heatmap illustrating the protein IDs of the top 20 identified proteins in pure HS I and HS II from mass spectrometry-based proteomics analysis (with intensities normalized to HSA). Gray color indicates that the identified protein is not included in the top 20 intensity proteins identified. (B) Heatmap illustrating the protein ID of the top 20 intensity proteins identified in HS I and HS II samples from mass spectrometry-based proteomics analysis, removing human serum albumin (protein ID: HSA) from analysis. Intensity values are normalized to IGHG1 intensity for HS I and PSMA4 for HS II. Gray color indicates that the protein is not included in the top 20 intensity proteins identified. (C) Heatmap illustrating the protein ID of the top 20 intensity proteins identified in the native protein corona of PtNP trastuzumab (unblocked) conjugates incubated in HS I and HS II. Intensity values are normalized to APOA1 intensity for both samples. (D) Heatmap illustrating the protein ID of the top ten intensity proteins identified in the native protein corona of PtNP trastuzumab conjugates incubated in human serum samples (P1–29). In each case, the observed intensities were normalized to the highest intensity protein identified. Gray color indicates that the protein is not included in the top ten intensity proteins identified.

To further probe protein fouling on PtNP surfaces, we characterized the composition of the formed native protein corona using the 29 individual human serum samples. The sample preparation was performed according to the aforementioned methodology. For each human serum sample, the identified proteins demonstrating the ten highest intensities and abundance were extracted and compared. The intensity values were normalized to the highest intensity protein for each sample to enable comparison of relative protein abundance (Figure 3D). The highest intensity proteins identified in the native protein corona varied between the human serum samples used (Table S9), and included: HSA, APOA1, APOC3, FN1, and IGHG1. While the identity of the highest intensity protein differed between samples, the highest intensity proteins demonstrated good agreement between the 29 samples (and HS I and HS II trial samples, Figure 3D). This includes the presence of human serum albumin (protein ID: HSA), apolipoprotein A1 (protein ID: APOA1), IgG (protein IDs: IGHG, IGKC), and kininogen-1 (protein ID: KNG1) in all samples, consistent with the findings from HS I and HS II pooled serum samples. The number of unique peptides (from the protein identification workflow) is shown for the top ten identified proteins (Tables S9–S11). Proteins with one unique peptide were excluded from the analysis, except for the protein with ID IGHV3OR16-9, due to its association with trastuzumab (Figure S10). Further, fibronectin (protein ID: FN1) and apolipoprotein C3 (protein ID: APOC3) were present in all but one sample, with FN1 demonstrating the highest intensity in 10 of the 29 samples (Table S9). Previous work using AuNPs has demonstrated that human serum albumin and apolipoprotein A1 are highly abundant in the native protein corona, which has downstream consequences on LFA performance.^63^ The consequences of fibronectin and kininogen-1 in the native protein corona have yet to be explored for in vitro diagnostic applications. However, for PEGylated liposomes, FN1 and KNG1 abundance in the protein corona increased on extended dynamic exposure.^71^

It was previously noted that varying total cholesterol concentration in the human serum samples was not the only source of interference in the LFA (Figure S8). Characterization of the native protein corona revealed that apolipoproteins are found to be highly abundant across the human serum samples, including apolipoproteins associated with both high-density lipoproteins (i.e., APOA1) and low-density lipoproteins (i.e., APOB and APOC3). While total cholesterol concentration was found not to be the sole cause of LFA performance variation, lipoproteins play a critical role in native protein corona formation around PtNPs, which may in turn modulate the functionality of the detection probes. The composition of the native protein corona of PtNP trastuzumab conjugates was distinct from the most abundant proteins present in human serum. In human serum and plasma, highly abundant proteins include albumin and globulins (including IgGs).^72–73^ The native protein corona formed around PtNPs demonstrates that the highest intensity proteins were not dictated by abundance alone.

### BOLD Methodology: Engineering an Idealized Corona

With an understanding of the native protein corona composition, we hypothesized that preforming a rationally selected, engineered protein corona could reduce the observed LFA interference and increase assay robustness. To enable control over the composition of the preformed protein corona, we employed isolated human proteins as blocking agents (i.e., in place of the previously used β-casein blocking agent). The protein identity was selected based on the proteomics analysis (Figure 3D), selecting proteins that were abundant across several human serum samples. Initially, we utilized individual human proteins as blocking agents (Figure 4A), where the following proteins were selected: HSA, apolipoprotein A1 (APOA1), apolipoprotein B (APOB), apolipoprotein C3 (APOC3), IgG, kininogen-1 (KNG1), and fibronectin (FN1). The proteins selected were utilized owing to their widespread identification across all 29 patient samples. In addition to their identification in the protein corona, the apolipoproteins (APOA1, APOB, APOC3) were included due to their role in cholesterol transport.^74^ Other proteins identified in the top ten intensity proteins, including complement component 3 (protein ID: C3), were excluded from additional analysis due to the lack of identification across all 29 samples.

**Figure 4:**
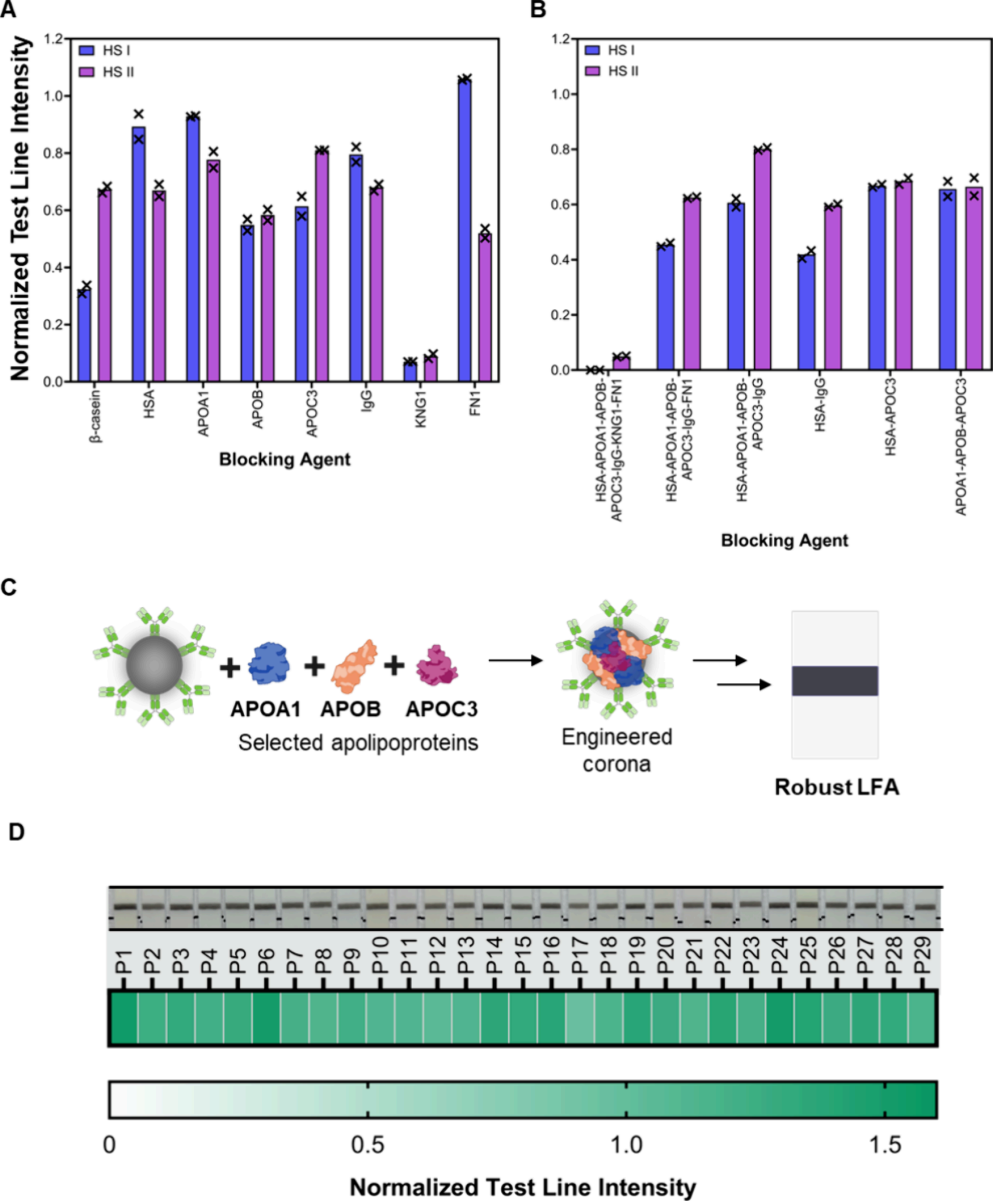
Assessment of performance variation using an engineered, preformed corona using identified proteins from the native protein corona analysis. (A) Assessment of LFA test line intensity using isolated proteins as blocking agents to produce an engineered corona. LFA performed with spiked HS I and HS II, *n* = 2. The use of β-casein as a blocking agent demonstrates the previously observed discrepancy in generated LFA test line intensity. (B) Assessment of LFA test line intensity using rationally selected combinations of isolated proteins as blocking agents to produce an engineered corona. LFAs performed with spiked HS I and HS II, *n* = 2. (C) Schematic of an engineered apolipoprotein preformed around PtNP-trastuzumab detection probes. The engineered corona enables standardization in the observed LFA performance. The composition of the engineered corona is established from characterization of the native protein corona. (D) Heatmap of extracted LFA test line intensity for individual patient samples (P1–29) using PtNPs with APOA1-APOB-APOC3 engineered corona, with representative images depicted above. Normalized intensity represents the mean value, *n* = 3.

We used HER2-biotin spiked HS I and HS II as sample matrices to screen the performance of PtNPs with distinct preformed protein coronas. No nonspecific binding was observed when using HS I and HS II (without antigen spiking) (Figure S11). On spiking HS I and HS II with HER2-biotin, the isolated protein blocking agents produced differing performances in terms of absolute test line intensity and comparative performance between HS I and HS II (Figures 4A, S12, and S13). When using FN1 as a blocking agent, the disparity in signal generated with HS I and HS II was highest, originating from reduced PtNP conjugate stability, as observed by visible aggregation. When HSA, APOA1, and IgG were used as individual blocking agents, the observed test line intensity was higher for HS I, compared to HS II (at equal spiked antigen concentrations). In contrast, APOC3 demonstrated the opposite trend. However, for all blocking agents, the observed discrepancies were reduced compared to the use of β-casein as a blocking agent (Figure S13A). PtNP conjugates blocked with KNG1 produced weak test lines that were barely visible for both HS I and HS II (Figure S12). The reduced test line intensity suggests that KNG1 is a key interfering substance that modulates the LFA performance. The origin of the observed interference is explored experimentally and through the employment of Molecular Dynamics (MD) simulations (vide infra).

Beyond using these proteins as discrete blocking agents, we explored the use of combinations of isolated proteins to preform an engineered corona. This included: 1) use of all seven blocking agents (HSA-APOA1-APOB-APOC3-IgG-KNG1-FN1), (2) use of all seven blocking agents minus KNG1 (HSA-APOA1-APOB-APOC3-IgG-FN1), (3) use of all seven blocking agents minus KNG1 and FN1 (HSA-APOA1-APOB-APOC3-IgG), (4) HSA-IgG, (5) HSA-APOC3, and (6) APOA1-APOB-APOC3 (Figure 4B). As with previous experiments, no nonspecific binding was observed on the use of HS I and HS II without the HER2-biotin antigen (Figure S14). When using all seven isolated proteins simultaneously, we observed a weakly visible test line for HER2-biotin spiked HS I and HS II, consistent with the use of KNG1 in isolation (Figures 4B and S15). This finding indicates preferential binding of KNG1 over the other six isolated proteins, to inhibit PtNP conjugate performance. On removal of KNG1 and use of the remaining six isolated proteins, we observed a restoration of LFA performance, whereby no false-negative signals were observed. The return of PtNP activity suggests that KNG1 plays a critical role in dictating PtNP detection probe performance. This finding may underpin the observed differences in the use of individual human serum samples. KNG1 is a histidine-rich protein involved in blood coagulation.^75,76^ While the adsorption mechanism of KNG1 to PtNPs has not been previously reported, histidine-rich moieties have been shown to favor noble metal nanoparticle adsorption.^75,76^ It is hypothesized that the histidine residues may promote the binding of KNG1 to PtNP surfaces, as explored below through MD simulations. The use of HSA-APOC3 as combined blocking agents was selected owing to the competing performance trends when used in isolation (Figures 4A and S13). When used together, we observed a standardized test line intensity between HS I and HS II, generating comparable test line intensities between the two serum samples. Further, we employed an apolipoprotein engineered corona (APOA1-APOB-APOC3), due to the three apolipoproteins (APOA1, APOB, or APOC3) producing competing effects on LFA performance when used in isolation (Figure 4A). The combined use of apolipoproteins was selected due to the synergistic biochemical interactions between apolipoproteins for in vivo cholesterol transport.^74^ Beyond this, APOA1, APOB, and APOC3 have contrasting physicochemical properties, including molecular weight and isoelectric points. Specifically, the combined use of varying molecular weight surface passivating agents is hypothesized to increase surface coverage, reducing the area of unfunctionalized PtNP surface, which can then interact with serum proteins. The apolipoprotein-engineered corona was therefore selected for further evaluation, owing to the presence of apolipoproteins in the native protein corona and their hypothesized synergistic interactions for effective surface passivation.

The combination of apolipoproteins (i.e., APOA1-APOB-APOC3) to preform an engineered corona was successful in minimizing observed variation in the signal generated with spiked HS I and HS II (Figure S15). The resultant engineered corona PtNP detection probe demonstrated a single, narrow size distribution, demonstrating no aggregation on functionalization (Figure S16). To assess the ability of an engineered apolipoprotein corona to improve LFA robustness, we explored the use of apolipoprotein blocking agents with the aforementioned 29 individual patient samples. We utilized the three isolated apolipoproteins as blocking agents during PtNP functionalization, preforming an engineered and idealized protein corona (Figure 4C). The performance in the developed LFA was then assessed by use of patient samples alone (negative samples, Figure S17) and after spiking of samples with HER2-biotin (positive samples, Figure S18) (Figure 4D). Compared to the use of β-casein as a blocking agent (Figure 3A), the APOA1-APOB-APOC3 preformed corona PtNPs demonstrated reduced aggregation at the base of the LFA strips (Figure S18). On the extraction of test line intensity, we observed an increase in test line intensities, compared to the use of β-casein as a blocking agent (Figures 2E and 4D). Further, all patient samples produced visually detectable test line intensities, with observable increases in the test line intensities (mean normalized test line intensity of 1.26, compared to 0.30 for conventional β-casein blocking) and no false-negative results. The engineered apolipoprotein corona was therefore able to restore LFA performance when used with samples that previously produced false-negative results (P3 and P26). Between individual patient samples, the variation in LFA test line intensity was significantly diminished (CV of 0.12 for engineered corona vs 0.58 for conventional β-casein blocking). Through engineering a preformed, idealized protein corona, we demonstrated increased LFA robustness for use with individual patient serum samples. Further, to move toward translating the apolipoprotein-engineered corona, functionalized PtNPs were dried prior to use (Figure S19). The apolipoprotein conjugates were resuspended prior to use, demonstrating initial feasibility for use without cold storage.

### Interactions of Serum Proteins with PtNPs

To explore the observation that highly abundant serum proteins did not solely dictate native protein corona composition, we established plate-based assays to probe the interactions of isolated human proteins with PtNPs (Figure 5A). We explored the ability of the aforementioned isolated human proteins (HSA, APOA1, APOB, APOC3, IgG, KNG1, and FN1), as well as β-casein, owing to its popular use as a blocking agent with PtNPs,^7–9,65^ and trastuzumab as the detection antibody in the model LFA. The plate-based assays utilized isolated proteins immobilized onto high-binding polystyrene well-plates, varying in molar concentration to estimate concentration-dependent binding affinity. Unfunctionalized PtNPs were then incubated in the protein-functionalized wells. A detectable signal was generated through the use of the intrinsic peroxidase-mimicking activity of PtNPs on the addition of peroxidase substrates (3,3′,5,5′-tetramethylbenzidine (TMB)).^7,9^ This is in contrast to the use of PtNPs in the aforementioned LFA, whereby PtNPs were utilized solely as high-contrast optical detection probes, without employment of catalytic signal amplification. The signal-to-noise ratio (SNR) was generated using an unfunctionalized well plate as a control (i.e., noise), as shown in Figure 5B.

**Figure 5:**
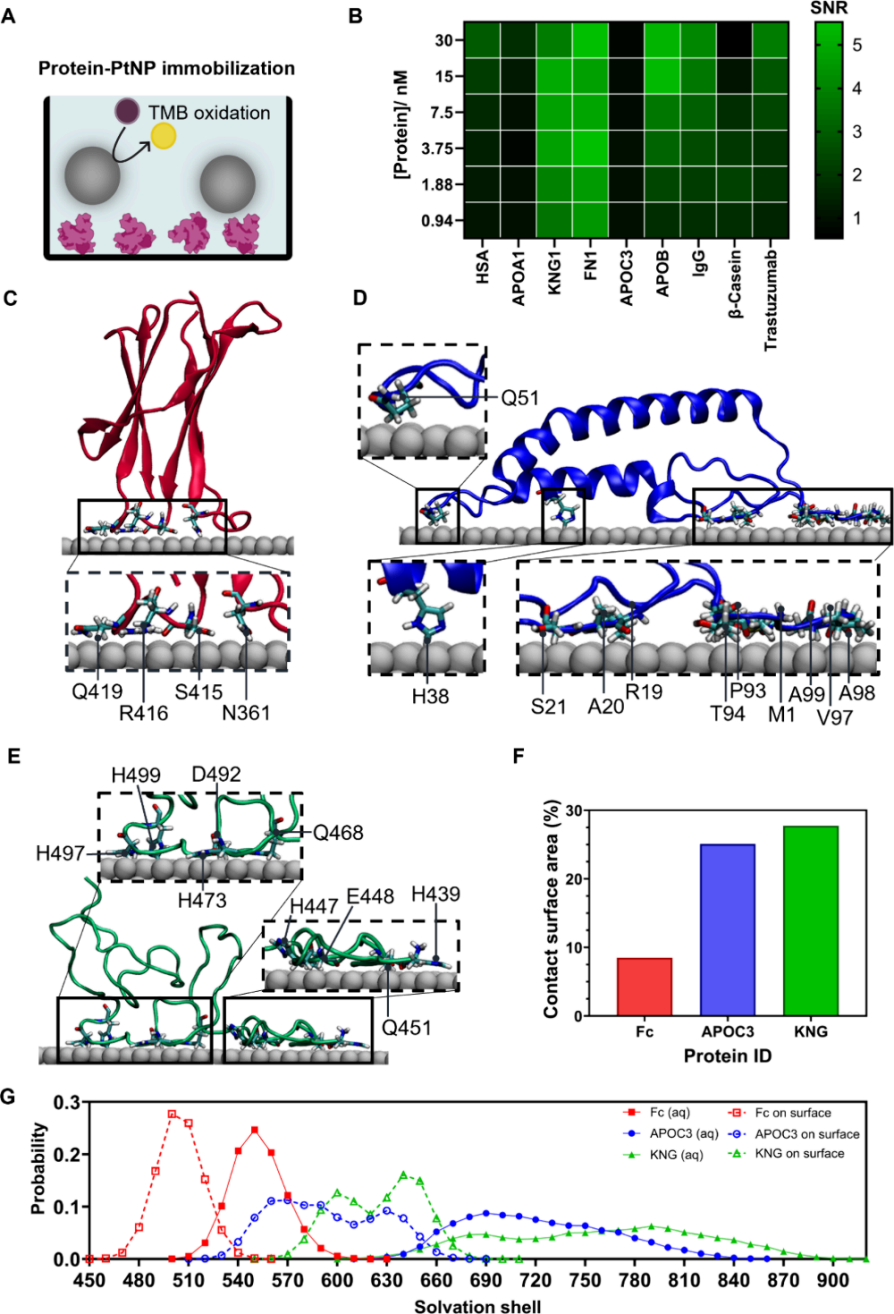
Analysis of protein binding affinity to PtNPs. (A) Schematic of plate-based assays, with immobilized isolated proteins and addition of PtNPs. (B) Signal-to-noise (SNR) ratio of PtNPs incubated with relevant isolated proteins, *n* = 3. Signal is produced with immobilized proteins at varying molar concentrations, and noise is produced with no protein addition to the well-plate. (C–E): Exemplar MD simulated configurations of (C) Fc monomer, (D) APOC3, and (E) the surface binding region of KNG1 on the surface of Pt(111). The inset pictures illustrate the contacts of residues on Pt(111). Color codes: Pt(111)- gray; carbon atoms- cyan; hydrogen atoms- white; oxygen atoms- red; nitrogen atoms- blue. (F) Contact surface area of proteins on the surface on Pt(111). (G) Distribution of the water molecules surrounding the proteins. Filled symbols represent proteins in aqueous (aq) solution and open symbols on the Pt(111) surface.

The SNR produced using immobilized KNG1 and FN1 was approximately concentration independent, producing SNR significantly above 1 for all concentrations evaluated (ca. 1–30 nM). This indicates that KNG1 and FN1 are effectively able to immobilize bare PtNPs through high-affinity electrostatic interactions. At low concentrations of immobilized protein (i.e., 1.88 nM), the SNRs with immobilized KNG1 and FN1 are significantly higher than for immobilized trastuzumab. This suggests that KNG1 and FN1 bind to PtNP surfaces with high affinity, and could therefore replace immobilized detection antibodies. In contrast, several proteins (HSA, APOB, IgG, and trastuzumab) demonstrated concentration-dependent binding to PtNPs. Of these proteins, APOB showcased the highest SNR at reduced immobilized protein concentrations. Interestingly, both APOA1 and APOC3 demonstrated SNRs of ca. 1 for the concentration ranges evaluated, signifying reduced ability to immobilize PtNPs in this format. These two proteins have lower molecular weights compared to the other proteins used (28 kDa for APOA1, 8.8 kDa for APOC3). We hypothesize that the low SNR may result from poorer protein immobilization onto the polystyrene well plate and protein denaturation on immobilization, leading to an inability to bind and immobilize PtNPs. However, these apolipoproteins have demonstrated functionality as blocking agents in LFAs (Figure 4A) and are present in the native protein corona (Figure 3D).

All-atom Molecular Dynamics (MD) simulations were employed to explore the observed differences in protein-PtNP immobilization and binding affinity (Figure 5A,B). Here, MD simulations investigated the binding of three proteins (the CH3 domain of the Fc region of human IgG, APOC3, and the surface binding region of KNG1 ^77^), selected based on their varying binding capacity to PtNPs in the plate-based assays (Figure 5B). The PtNP was modeled as a Pt(111) surface, the most energetically favorable crystallographic facet of platinum nanoparticles.^78^

Considering the human IgG Fc monomer, MD simulations identified a limited number of residues (specifically glutamine, arginine, serine, and asparagine) that facilitate adsorption onto the Pt(111) surface (Figures 5C and S20A). In contrast, APOC3 demonstrated a larger range of amino acid residues (including methionine, arginine, alanine, serine, histidine, glutamine, proline, threonine, and valine) that facilitated binding to the Pt(111) surface (Figures 5D and S20B). For KNG1, which demonstrates the highest degree of PtNP immobilization and produced negative LFA interference, a greater total number of residues were involved in interaction with the Pt(111) surface, dominated by histidine, glutamic acid, aspartic acid, and glutamine residues (Figures 5E and S20C).

Further, water molecule contacts and contact surface areas for each protein-Pt(111) surface interaction were calculated (Figure 5F,G). On adsorption onto the Pt(111) surface, the three proteins demonstrated a reduction in the number of surrounding water molecules, indicating displacement of bound water molecules. Previous work has suggested that the removal of bound water from the Pt(111) surface is energetically less favorable compared to the displacement of free water.^79,80^ However, the energy gained through protein–surface interactions could partially compensate for this energy cost. Moreover, the release of bound water increases entropy, which would likely contribute to the thermodynamic stabilization of the protein in its bound state. The calculated contact surface area demonstrated variation between the three selected proteins. The KNG1 surface binding region exhibited the highest surface contacts, whereby 28% of the total protein surface area contacted the Pt(111) surface. Likewise, APOC3 demonstrated considerable surface contact with the Pt(111) surface, calculated to involve 25% of the total protein surface. In contrast, the Fc monomer demonstrated the lowest surface contact area, where only 8.5% of the protein unit was in contact with the Pt(111) surface.

Moreover, the mobility of the protein residues was evaluated and quantified by comparing the root-mean-square-fluctuation (RMSF) of the protein on the surface to that in aqueous solution (Figure S21). On surface binding, both the Fc monomer and APOC3 were found to have a significant proportion of more mobile residues, demonstrated by residues with ΔRMSF values greater than 0 nm (Figure S21A,B). In contrast, KNG1 was found to contain significantly more rigid residues, with ΔRMSF values less than 0 nm (Figure S21C). This illustrates that the surface binding region of KNG1 is more stable when adsorbed onto Pt(111), compared to the other two proteins. It is hypothesized that this is due to the higher proportion of histidine residues in KNG1, which are involved in protein adsorption, contributing to favorable interactions with the Pt(111) surface.^81^ This is further supported by a prolonged residence time of surface binding, where 25 residues in KNG1 show a relatively high percentage (>50%) of residence time for the adsorption to the Pt(111) surface (Figure S22). Specifically, histidine residues, ranging from H443, H447, H453, H459, H463, H465, H469, and H493 were identified to have relatively high residence times (Figure S22C). Conversely, both the Fc monomer and APOC3 showed weaker adsorption to the Pt(111) surface, with no histidine residues observed to bind (Figure S22A,B). The Fc monomer showcased only four residues with a contact probability of >0.5 and a normalized residence time of >50% (Figure S22A), while APOC3 demonstrated 19 residues with a contact probability of >0.5 and a normalized residence time of >50% (Figure S22B). Overall, consistent with the experimental observations, the MD simulations indicate that the surface binding region of KNG1 binds favorably to the Pt(111) surface, whereas both APOC3 and the Fc monomer appear to form weaker and more highly mobile interactions.

## Conclusions

Here, we demonstrate that interference in LFAs can occur with the use of human serum samples from individual donors, leading to highly variable LFA test line intensities and the presence of false-negative results. This was demonstrated using samples spiked with a non-native antigen (HER2-biotin), using conventional detection probe functionalization methods. To address this, we developed an optimization workflow, based on bionano interface optimization (termed BOLD). In this method, we characterized the interface between PtNP detection probes (functionalized with trastuzumab detection antibodies) and the human serum samples to enable insight into the extent of protein fouling and native protein corona formation. We subsequently preformed an engineered corona to standardize protein fouling across all human serum samples tested. We identified that the composition of the engineered corona had direct consequences on LFA performance. Specifically, when using KNG1 as a blocking agent (individually and in combination with other isolated proteins), we observed a significant reduction in the LFA test line. This demonstrated that KNG1 was able to preferentially bind to the PtNP surface, leading to a reduction in PtNP conjugate performance. This finding is supported by MD simulations, illustrating that KNG1 adsorbs to PtNP surfaces through a variety of amino acid residues, leading to increased rigidity of residues when compared to their mobility in aqueous solution. We utilized combinations of isolated human proteins (such as HSA-APOC3, and APOA1-APOB-APOC3) to preform an engineered corona, resulting in standardization of LFA performance when using pooled human serum samples. Further, we engineered an idealized apolipoprotein corona (APOA1-APOB-APOC3 blocking proteins) for use alongside human serum samples from individual patients, which previously exhibited high levels of signal variation (CV of 0.58). The engineered apolipoprotein corona was successful in reducing signal disparity (CV of 0.12) between individual samples and resulted in a notable increase in the observed test line intensity.

Overall, this work provides an alternative approach for LFA optimization through modulation of the bionano interface. The BOLD method facilitates rational optimization of detection probes for use in LFAs, generating detection probes with an engineered preformed protein corona that is appropriate for its intended use. This includes the use of relevant isolated proteins for the desired complex biological sample, without generalizing between nanoparticle species and sample matrix. The BOLD methodology can be readily translated for use with other biofluids (for example, plasma and saliva) and detection probes (i.e., nanoparticle species functionalized with detection antibodies) for the detection of disease-specific biomarkers. This includes the use of conventional nanoparticle probes (i.e., AuNPs) and next-generation nanoparticle species (i.e., nanozymes). This approach paves the way for robust optimization, informed directly from characterization of the bionano interface.

## Materials and Methods

### Synthesis of Platinum Nanoparticles (PtNPs)

The synthesis of platinum nanoparticles (PtNPs) was performed according to previously reported protocols with slight modifications.^7–9^

Briefly, PtNPs (ca. 120 nm) were synthesized via the reduction of chloroplatinic acid hydrate onto the 15 nm AuNP seeds. Glassware (4 × 24 mL glass vial) was washed 3× with 10 mL of UPDW before PtNP synthesis. To each glass vial, 17.53 mL of ultrapure distilled water (UPDW, Invitrogen) was added, followed by 2.49 mL of 15 nm AuNP seed (2.49 nM, BBI Solutions). To the solution, 400 μL of 20 w/v% of poly­(vinylpyrrolidone) (PVP, MW 10 kDa, Sigma) was added. The solution was briefly vortexed and incubated without stirring for 5 min. To the polymer coated AuNP seed solution, 800 μL of l-Ascorbic acid (100 mg mL^–1^, Sigma) was added, followed by the addition of 800 μL of chloroplatinic acid hydrate (100 mM, Sigma). The resulting solution was briefly vortexed and immediately incubated at 65 °C without stirring for 45 min. The black PtNP solution was then cooled to room temperature (RT), and excess reagents were removed through three sequential washing cycles at 7000 rcf for 10 min with resuspension into UPDW. After the final wash step, PtNCs were resuspended in UPDW (300 pM) and stored at 4 °C before further use.

### Production of β-Casein Blocked PtNP Trastuzumab Detection Probes

Detection probes were produced via the adsorption of antibodies onto the surface of PtNPs. Briefly, 100 μL of PtNP solution (300 pM) was added to a 0.5 mL Protein LoBind Eppendorf, before the addition of 10 μL of conjugation buffer (100 mM carbonate buffer, pH 9). Antibody solution (1.80 μL, 1 mg mL^–1^, trastuzumab, Biosynth) was added at a ratio of 400:1 (antibody:PtNP). The resulting mixture was briefly vortexed and incubated at RT for 3 h under shaking (700 rpm). The PtNP trastuzumab conjugates were then blocked via the addition of 100 μL of blocking solution 2 w/v% β-casein [Sigma] in Dulbecco’s PBS (DPBS) and further incubation at RT for 1 h under shaking (700 rpm). Excess reagents were removed through 3× washing steps. Conjugates were centrifuged at 5000 rcf for 5 min to form a pellet, the supernatant removed, and conjugates resuspended in 200 μL of wash buffer (0.2 w/v% β-casein, 0.2 v/v% Tween20 in DPBS). After the final wash step, the conjugate was resuspended in wash buffer to a final concentration of 300 pM and stored at 4 °C.

### Operation of the Lateral Flow Assay (LFA)

All LFAs were performed by immersing a constructed LFA test strip (polystreptavidin, 1 mg mL^–1^, Mologic) into a 96-well clear flat-bottom polystyrene nonbinding surface microplate (Corning) containing: 50 μL sample (sample spiked with HER2-biotin, Sino Biological), and 5 μL of PtNP trastuzumab (300 pM). When the solution had fully wicked up the LFIA strip (ca. 10 min), the strip was photographed with an iPhone 15 camera and images processed using ImageJ.

### Preparation of Samples for RP-nLC/ESI-MS Analysis

Details of protein sample preparation for RP-nLC/ESI-MS analysis are outlined in the Supporting Information.

### RP-nLC/ESI-MS Protocol

Full details of the reverse phase-nano liquid chromatography/electrospray ionization-mass spectrometry (RP-nLC/ESI-MS) protocol can be found in the Supporting Information.

### RP-nLC/ESI-MS Data Analysis

MaxQuant (v2.6.7.0) was used to analyze the raw RP-nLC/ESI-MS data files. The data were run against the *Homo sapiens* UniProt FASTA file (proteome ID: UP000005640_9606).^82^ The following parameters were utilized: Trypsin/P digestion, maximum of 2 missed cleavages, oxidation (M) and acetyl (protein N-term) variable modifications, carbamidomethyl (C) fixed modification, inclusion of contaminants, protein false discovery rate (FDR) set at 0.01 (high confidence).

### Production of PtNP Trastuzumab Conjugates with Engineered Protein Corona

Engineered protein corona PtNP trastuzumab conjugates were produced via the adsorption of antibodies and isolated human proteins onto the surface of PtNPs. Briefly, 100 μL of PtNP solution (300 pM) was added to a 0.5 mL Protein LoBind Eppendorf, before the addition of 10 μL of conjugation buffer (100 mM carbonate buffer, pH 9). Antibody solution (1.80 μL, 1 mg mL^–1^, trastuzumab, Biosynth) was added at a ratio of 400:1 (antibody:PtNP). The resulting mixture was briefly vortexed and incubated at RT for 3 h under shaking (700 rpm). The PtNP trastuzumab conjugates were then blocked via the addition of isolated human proteins. The ratio of isolated human protein to PtNP was calculated based on molecular weight. Further details are described in the Supporting Information. Excess reagents were removed through 3x washing steps. Conjugates were centrifuged at 5000 rcf for 5 min to form a pellet, the supernatant removed, and conjugates resuspended in 200 μL of wash buffer (DPBST). After the final wash step, the conjugate was resuspended in wash buffer to a final concentration of 300 pM and stored at 4 °C.

### PtNP Plate-Based Interaction Assays

Isolated proteins (HSA; APOA1; APOB; APOC3; IgG; KNG1; FN1; β-casein) were diluted in 50 mM carbonate buffer, pH 9.6 (varying molar concentrations). 100 μL of diluted protein was added to the appropriate wells of a high-binding polystyrene 96-well plate (Corning). The solutions were incubated in the wells overnight at 4 °C. The wells were washed 3x with DPBST. 100 μL of PtNPs (unfunctionalized) were added to the corresponding wells at a concentration of 2 pM in DPBST. The PtNP solution was incubated in the wells for 15 min. The wells were washed 3x with DPBST. 100 μL of 1-Step TMB ELISA Substrate (Thermo Fisher) was added to each well and left to react for 15 min. The reaction was stopped by the addition of 50 μL of 4 M H_2_SO_4_. The absorbance at 450 nm was measured using a SpectraMax M5 microplate reader (Molecular Devices).

### Brief Computational Details

The all-atom MD simulations were performed in triplicate for each system using GROMACS 2021.4.^83,84^ The CHARMM36m force field was used to model all proteins in aqueous solution, while the INTERFACE force field was employed to characterize the intermolecular interactions between the proteins and the surface of Pt(111).^85,86^ Simulations for the protein–surface systems were run for 500 ns, with a time step of 2 fs. All proteins in aqueous solution were simulated for 200 ns simulations with a time step of 2 fs. Please refer to the Supporting Information for more detailed computational methodology.

### Simulation Data Analysis

Simulations were evaluated on the final 100 ns of equilibrated trajectories, with details on data visualization and analysis adapted from previous studies.^87,88^ The cutoff distance of water molecule count contacts is 0.35 nm, while the contact surface area was calculated by subtracting and normalizing the solvent-accessible-surface-area (SASA) values of the protein on the Pt(111) surface from the SASA of the bare protein. The cutoff distance for the protein–surface interactions was set at 0.4 nm.

### Oxford Biobank Human Serum Samples

Patient samples from 29 individual donors were received from Oxford Biobank, following standard collection methods. Serum samples were selected based on total cholesterol levels, with samples classified as < first, 45–55th, and >99th percentile. We thank the volunteers from the Oxford Biobank (www.oxfordbiobank.org.uk) for their participation in this recall study (Research Ethics Committee reference: 23/SC/0411). All participants gave informed consent.

## Supplementary Material



## Notes

Characterization of PtNPs and PtNP conjugates, photographs of LFA test strips, proteomics analysis of isolated proteins, proteomics analysis of PtNP conjugates in HS I and HS II, protein corona purification methodology, MD simulations (PDF)
