# Supplementary material for: Bionano Interface Optimization for Rational Lateral Flow Assay Development
Source: ACS Nano. 2026 May 1;20(18):13897–912. doi: 10.1021/acsnano.6c04136 (PMC13173653; doi:10.1021/acsnano.6c04136)
Supplement: Supplementary file 1 [file nn6c04136_si_001.pdf]

Supporting Information

Bionano Interface Optimization for Rational Lateral Flow Assay Development

Christy J. Sadler[1,2,3], Maya Miller[1,2], Kevion K. Darmawan[4], Jan P. Sandler[1,2,3,5], Ho-Cheung Ng[1,2,3], André Shamsabadi[1,2,3], Adam Creamer[1,2,3], Carol V. Robinson[2,6], Irene Yarovsky*[4], Molly M. Stevens*[1,2,3]

[1]Department of Physiology, Anatomy and Genetics, Department of Engineering Science, University of Oxford, OX1 3QU, United Kingdom

[2]Kavli Institute for Nanoscience Discovery, University of Oxford, OX1 3QU, United Kingdom

[3]Department of Materials, Department of Bioengineering, Institute of Biomedical Engineering, Imperial College London, London, SW7 2AZ, United Kingdom

[4]School of Engineering, RMIT University, Melbourne, VIC 3001, Australia

[5]Research Complex at Harwell, Harwell Science and Innovation Campus, Didcot, Oxfordshire OX11 0FA, United Kingdom

[6]Department of Chemistry, University of Oxford, Oxford, OX1 3TA, United Kingdom

*Corresponding authors: Irene Yarovsky (irene.yarovsky@rmit.edu.au) and Molly M. Stevens (molly.stevens@dpag.ox.ac.uk)

Contents:

Supporting Information Figures: *p02*

Supporting Information Experimental Section: *p27*

Supporting Information Computational Section: *p33*

Supporting Information References: *p37*

Supporting Information Figures:

Table S1: Biochemical analysis of HS I and HS II pooled human serum samples (S1-100 mL, Millipore).

	**HS I**	**HS II**
Lot Number	3986164	4130095
Glucose/ mg dL^{-1}	77	70
BUN/ mg dL^{-1}	12	8
Creatinine/ mg dL^{-1}	0.9	0.7
Sodium/ mmol L^{-1}	136	135
Potassium/ mmol L^{-1}	5.4	4.1
Chloride/ mmol L^{-1}	99	101
Calcium/ mg dL^{-1}	7.6	7.2
Phosphorus/ mg dL^{-1}	3.1	3.2
Uric Acid/ mg dL^{-1}	4.7	4.3
Total Protein/ g dL^{-1}	5.8	5.8
Albumin/ g dL^{-1}	3.4	3.6
Globulin/ g dL^{-1}	2.2	2.0
Total Bilirubin/ mg dL^{-1}	0.32	0.30
Alkaline Phosphatase/ IU L^{-1}	51	45
LDH/ IU L^{-1}	128	128
AST/SGOT/ U L^{-1}	17	16
ALT/SGPT/ U L^{-1}	8	8
GGTP/ IU L^{-1}	20.1	18.1
Osmolarity-Serum (calc)/ mOsm kg^{-1}	290	302.6
Ionized Calcium (calc)/ mg dL^{-1}	3.7	3.5
Total Iron/ µg dL^{-1}	76	76
Cholesterol/ mg dL^{-1}	134	127
Triglycerides/ mg dL^{-1}	73	42
pH	7.2	7.2
Preservatives	None	None

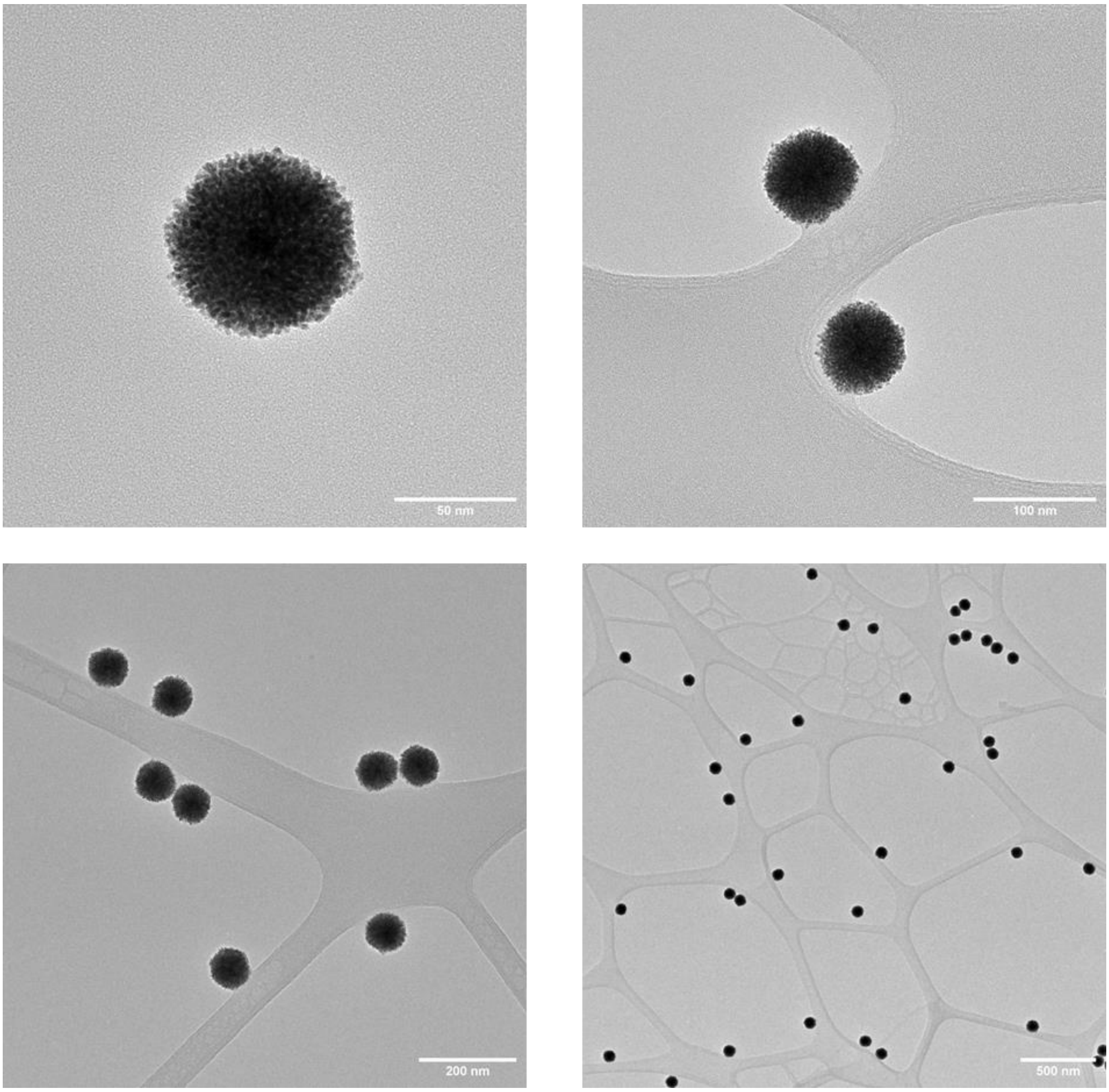

Figure S1: Transmission electron microscopy (TEM) micrographs of synthesized bare PtNPs. Four distinct regions on the grid are visualized at varying magnification factors.

Table S2: Characterisation metrics (Zeta Potential, Z-average and PDI) of bare PtNPs. Characterisation of bare PtNP stability and protein fouling on incubation with HS I and HS II, measured by DLS. Data is reported as mean ± SD, n = 3.

**Sample ID**	**Zeta Potential/ mV**	
Bare PtNP	-52.4 ± 0.12	
**Sample ID**	**Z-Average/ nm**	**PDI**
Bare PtNP	102.7 ± 1.49	0.026 ± 0.014
Bare PtNP + HS I	131.1 ± 0.32	0.113 ± 0.024
Bare PtNP + HS I t = 15 min	133.2 ± 1.90	0.105 ± 0.015
Bare PtNP + HS I t = 30 min	135.0 ± 1.02	0.131 ± 0.017
Bare PtNP + HS I t = 60 min	134.1 ± 1.50	0.088 ± 0.015
Bare PtNP + HS II	127.2 ± 0.35	0.078 ± 0.020
Bare PtNP + HS II t = 15 min	129.0 ± 0.87	0.087 ± 0.008
Bare PtNP + HS II t = 30 min	129.4 ± 0.46	0.089 ± 0.017
Bare PtNP + HS II t = 60 min	135.4 ± 2.84	0.095 ± 0.004

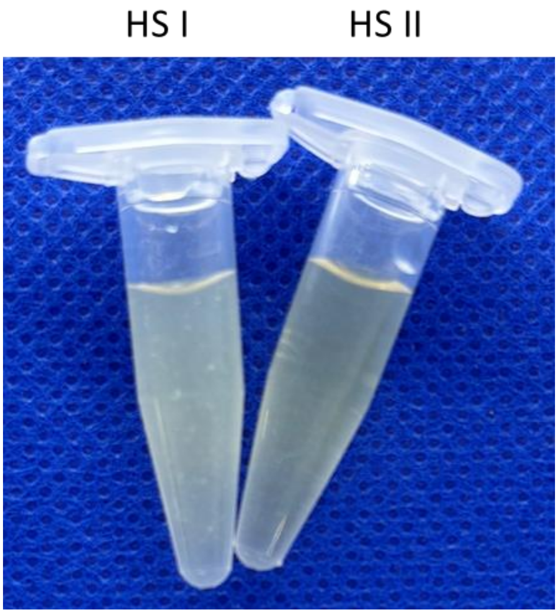

Figure S2: Photograph of HS I (left) and HS II (right), illustrating varying turbidity between the two pooled human serum samples.

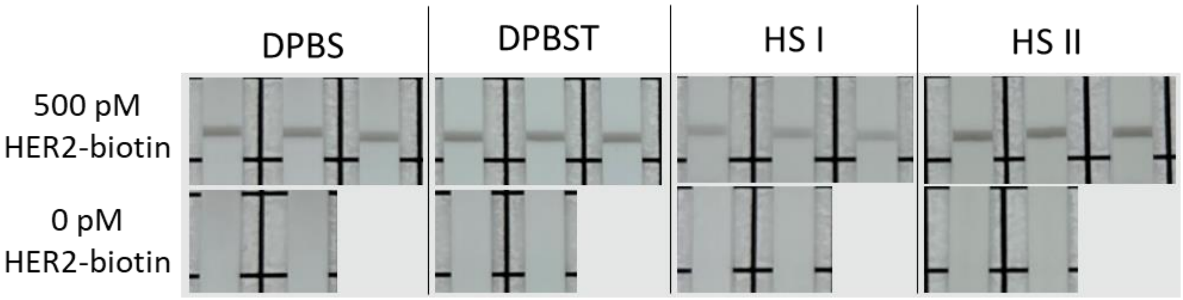

Figure S3: Photographs of LFA test strips used with spiked sample matrices (DPBS, DPBST, HS I and HS II). Top panel: LFA test strips used with 500 pM HER2-biotin (positive sample), n = 3. Bottom panel: LFA test strips used with 0 pM HER2-biotin (negative sample), n = 2.

A

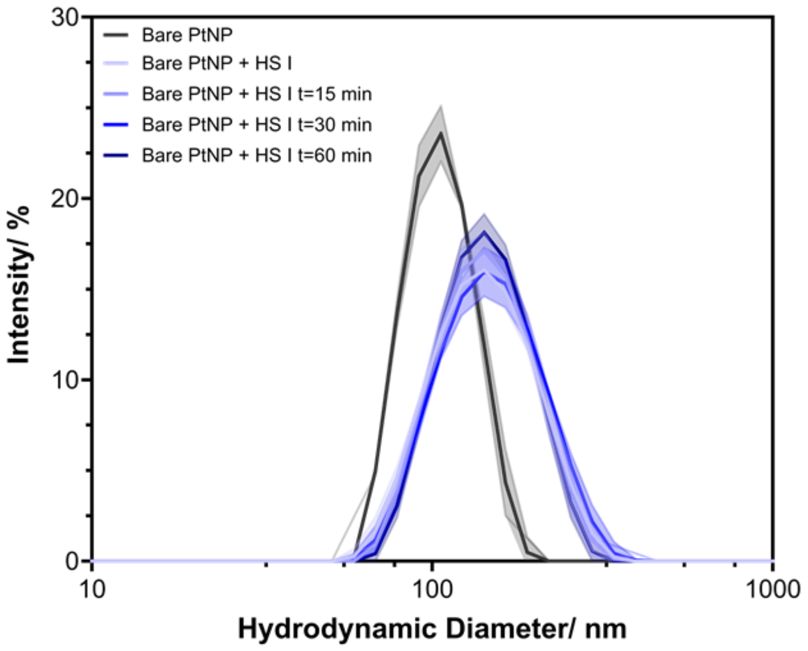

B

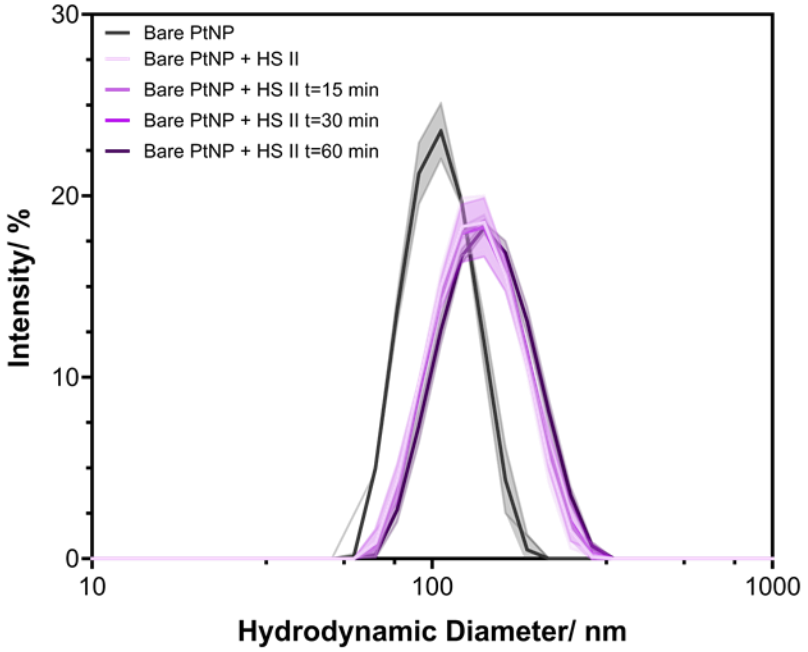

Figure S4: Assessment of change in hydrodynamic diameter on incubation of bare PtNPs with HS I (A) and HS II (B) over four incubation periods (initial, 15-, 30-, and 60-min) using DLS. The size distribution of bare PtNPs is shown in gray. Data plotted as mean ± SD, n = 3.

Table S3: Performance metrics (Z-average and PDI) to assess protein fouling on bare PtNPs that are incubated with human serum and subsequently washed, measured by DLS. Data is reported as mean ± SD, n = 3.

**Sample ID**	**Z-Average/ nm**	**PDI**
Washed bare PtNP + HS I t = 15 min	137.4 ± 2.46	0.080 ± 0.005
Washed bare PtNP + HS II t = 15 min	137.6 ± 1.15	0.085 ± 0.014

Table S4: Performance metrics (Z-average and PDI) to assess PtNP trastuzumab stability and protein fouling on incubation with HS I and HS II, measured by DLS. Data is reported as mean ± SD, n = 3.

**Sample ID**	**Z-Average/ nm**	**PDI**
PtNP Trastuzumab	125.9 ± 2.14	0.094 ± 0.022
PtNP Trastuzumab in HS I	134.9 ± 0.17	0.175 ± 0.002
PtNP Trastuzumab in HS II	136.2 ± 2.34	0.209 ± 0.023
PtNP Trastuzumab + β-casein	138.7 ± 3.00	0.075 ± 0.020
PtNP Trastuzumab + β-casein in HS I	145.0 ± 1.81	0.209 ± 0.017
PtNP Trastuzumab + β-casein in HS II	148.6 ± 5.07	0.018 ± 0.019

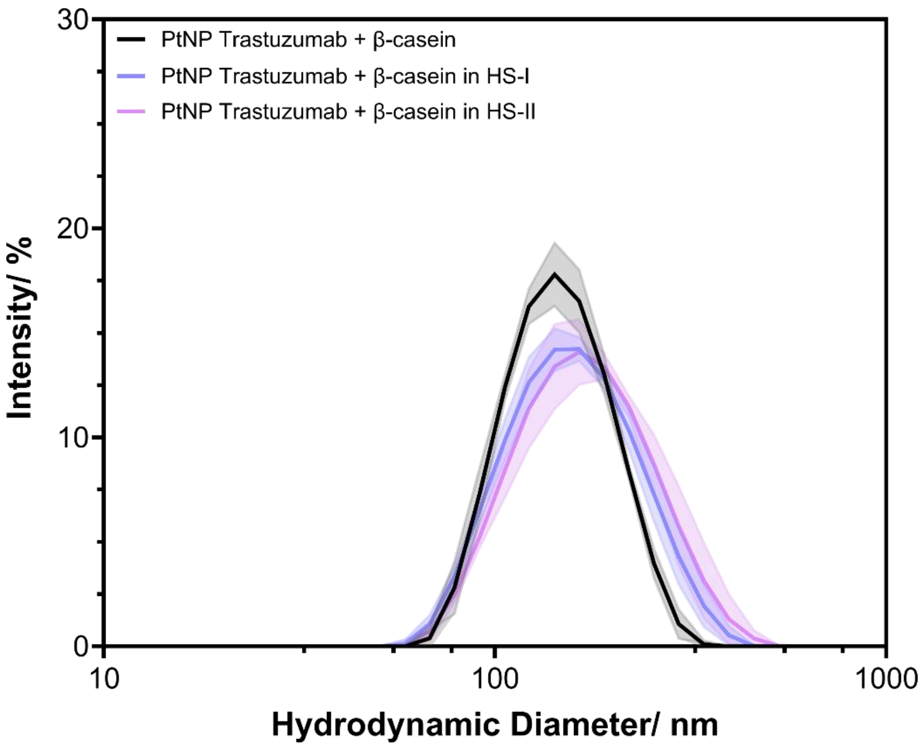

Figure S5: Assessment of change in hydrodynamic diameter on incubation of PtNP conjugates blocked with β-casein in HS I (blue) and HS II (purple) over 15-min incubation period using DLS. PtNP trastuzumab conjugates incubated in DPBS are shown in orange. Data plotted as mean ± SD, n = 3.

Table S5: Cholesterol levels from 29 human serum samples selected from the Oxford Biobank.

Sample ID	[Total Cholesterol]/ mg dL^{-1}	[Total Cholesterol] Percentile	[Low Density Lipoprotein, LDL]/ mmol L^{-1}
P1	194.50	45-50th	3.20
P2	387.10	>99th	7.85
P3	199.92	50-55th	3.24
P4	379.00	>99th	7.38
P5	116.78	<1st	1.18
P6	96.37	<1st	1.17
P7	309.74	>99th	5.73
P8	193.35	45-50th	3.22
P9	309.36	>99th	5.78
P10	367.36	>99th	7.24
P11	104.40	<1st	1.48
P12	193.35	45-50th	3.19
P13	328.70	>99th	5.91
P14	198.38	50-55th	3.19
P15	100.54	<1st	1.23
P16	96.67	<1st	1.41
P17	201.10	50-55th	3.18
P18	320.96	>99th	5.66
P19	324.80	>99th	6.31
P20	85.07	<1st	1.02
P21	201.10	50-55th	3.19
P22	201.10	50-55th	3.23
P23	201.10	50-55th	3.23
P24	355.76	>99th	6.88
P25	119.88	<1st	1.19
P26	110.60	<1st	1.24
P27	120.26	<1st	1.32
P28	338.75	>99th	6.30
P29	112.53	<1st	1.24

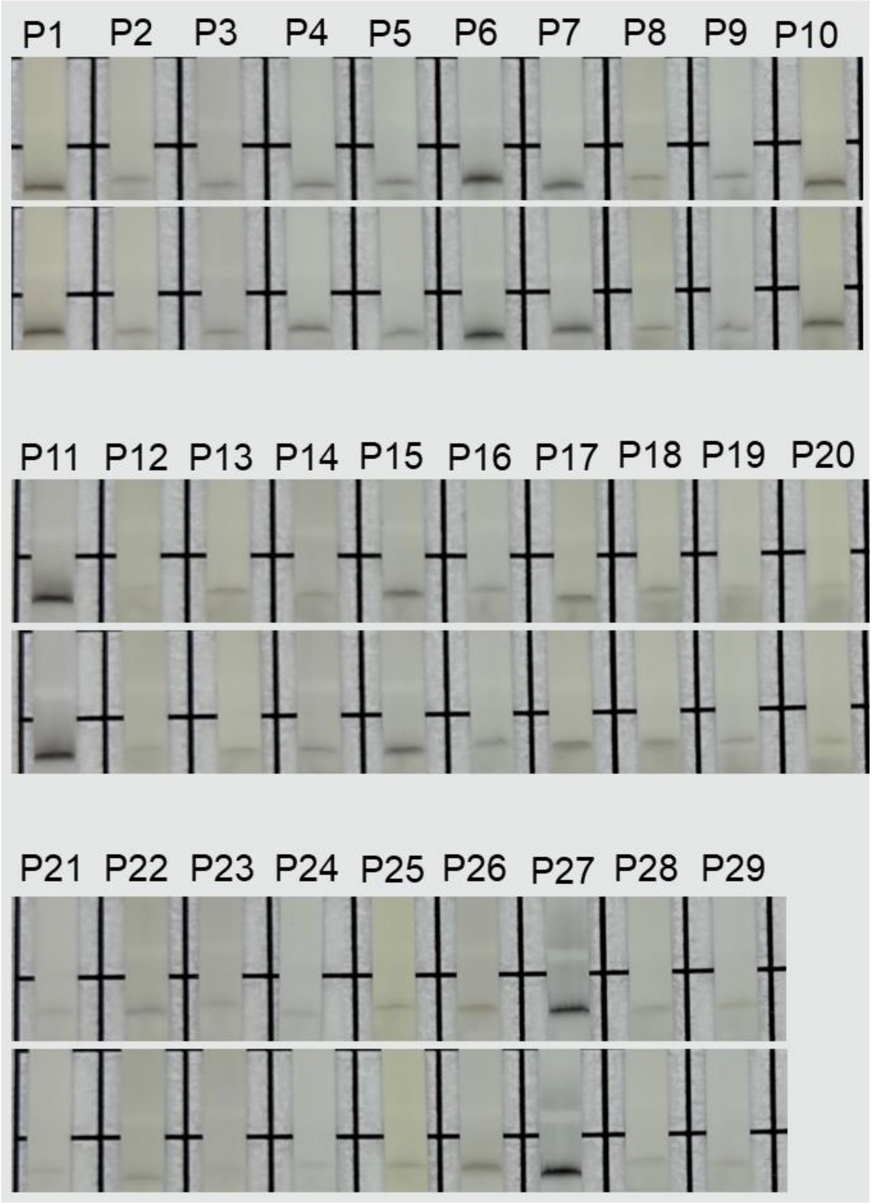

Figure S6: Photographs of LFA test strips using 29 human serum samples with 0 pM HER2-biotin (negative samples) (N = 29, n = 2).

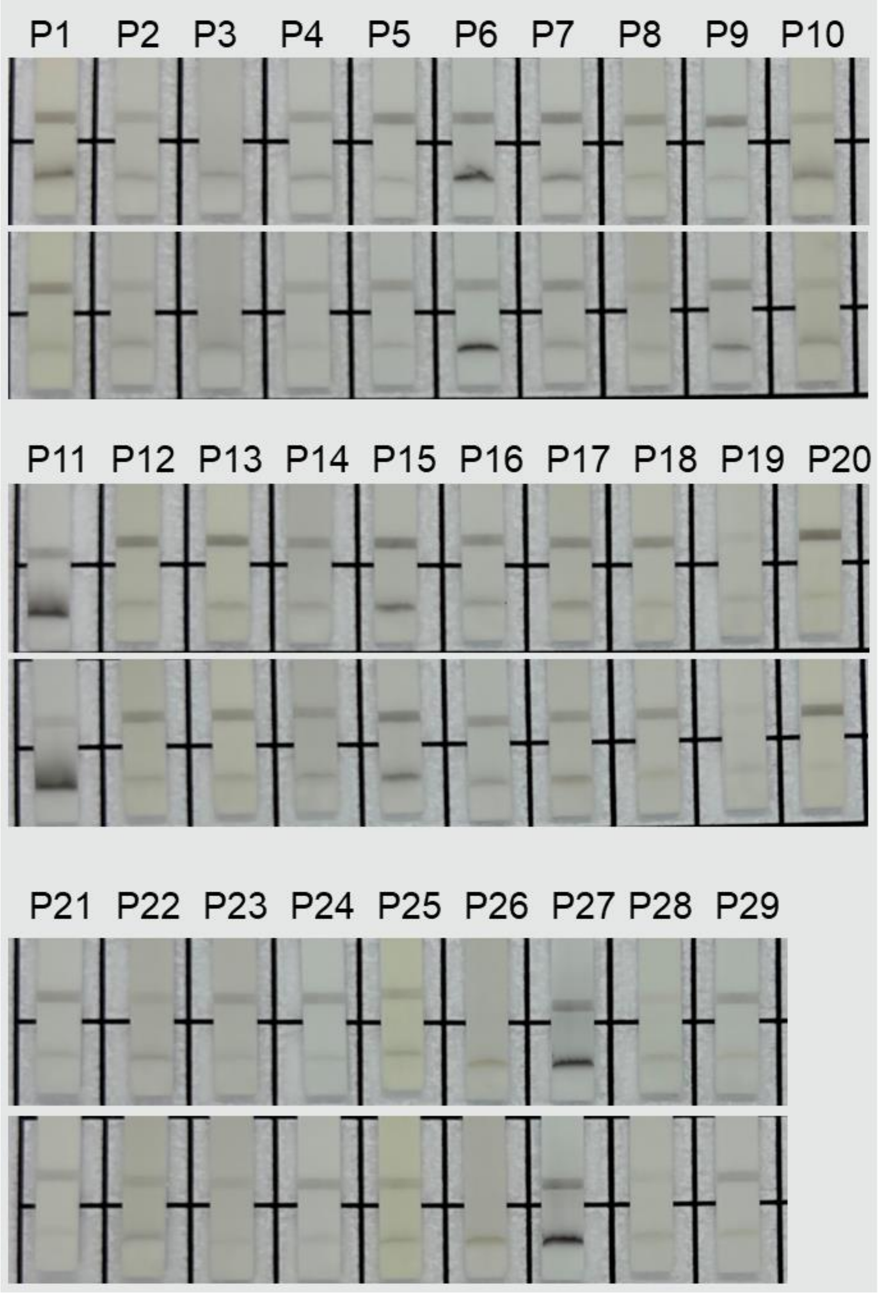

Figure S7: Photographs of LFA test strips using 29 human serum samples with 500 pM HER2-biotin (positive samples) (N = 29, n = 2). The human serum samples used with the LFA were spiked independently to produce the technical replicates.

Table S6: DLS of PtNPs incubated in P3 and P26 human serum samples to assess nanoparticle stability.

**Sample ID**	**Z-Average/ nm**	**PDI**
PtNP + P3 serum	139.6 ± 1.27	0.162 ± 0.007
PtNP + P26 serum	131.4 ± 0.62	0.172 ± 0.011

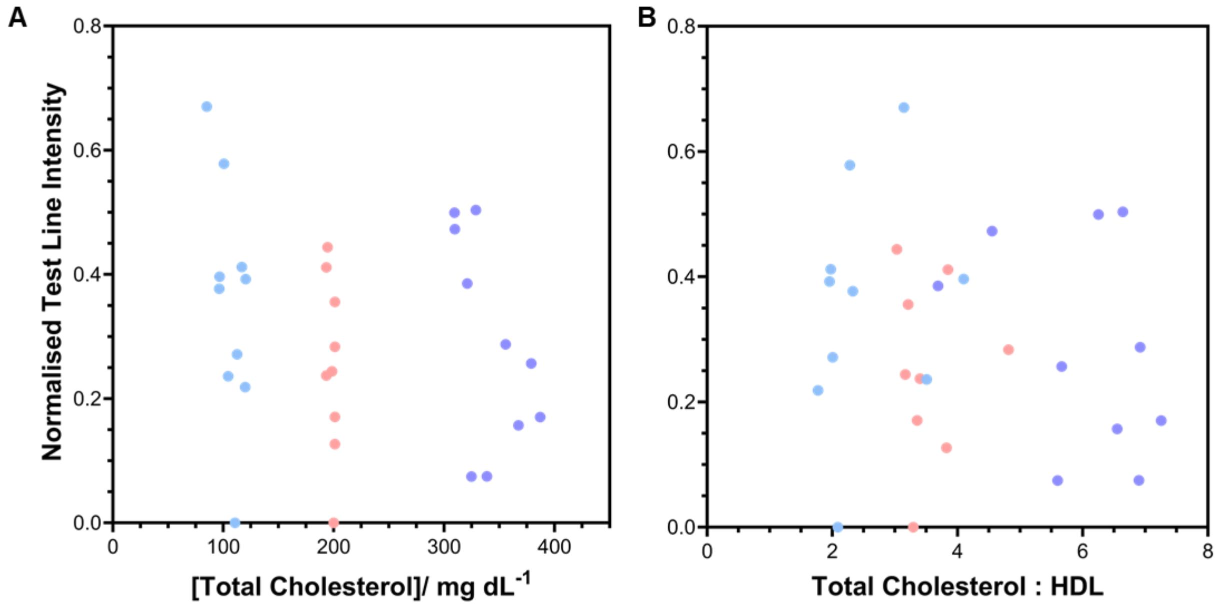

Figure S8: Assessment of cholesterol interference on LFA performance using human serum samples from 29 individual donors. A: Assessment of LFA test line intensity as a function of total cholesterol concentration. Data plotted as the mean test line intensity (N = 29, n = 2). B: Assessment of LFA test line intensity as a function of the ratio between total cholesterol and high-density lipoprotein (HDL) concentration. Data plotted as the mean test line intensity (N = 29, n = 2).

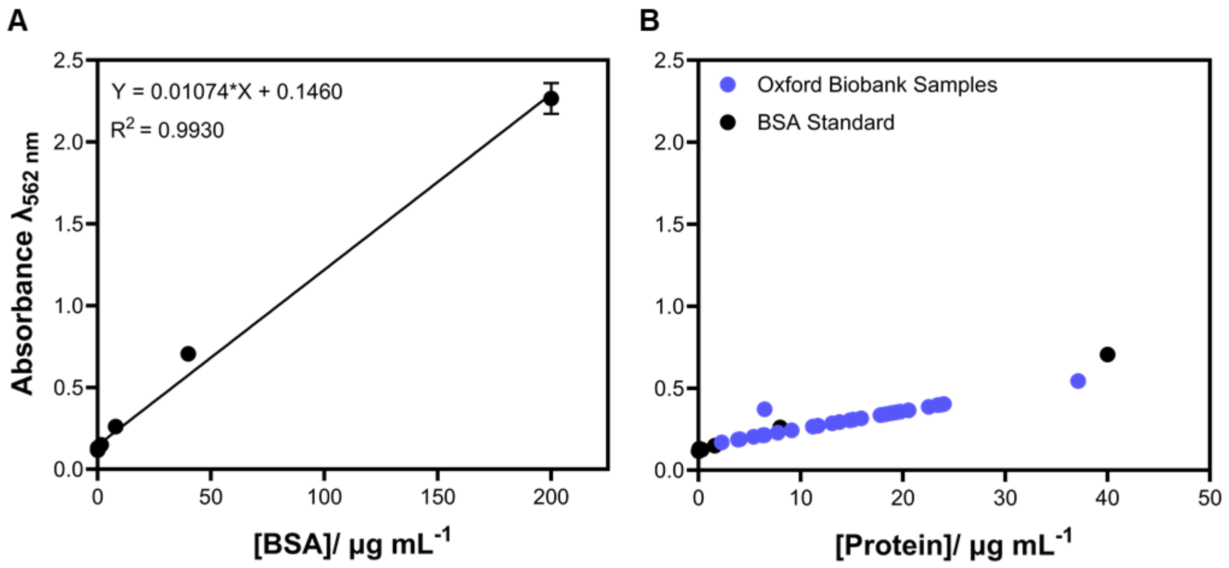

Figure S9: MicroBCA assay to determine protein concentration on final purification step prior to performing RP-nLC-ESI/MS experiments. A: Standard curve using bovine serum albumin (BSA), n = 3. B: Analysis of final wash supernatant of PtNP trastuzumab conjugate with human serum patient samples, n = 3.

Table S7: MicroBCA assay to determine protein concentration in supernatant of PtNP trastuzumab sample incubated in human serum. The remaining volume of supernatant in the PtNP trastuzumab sample incubated in human serum was 10 µL after the final purification step.

Sample ID	[Mean Protein]/ µg mL^{-1}	Mean Protein Mass in 10 µL/ µg
P1	15.12	0.1512
P2	15.91	0.1591
P3	17.94	0.1794
P4	14.80	0.1480
P5	6.28	0.0628
P6	20.54	0.2054
P7	19.35	0.1935
P8	23.99	0.2399
P9	7.75	0.0775
P10	4.05	0.0405
P11	5.38	0.0538
P12	18.74	0.1874
P13	18.29	0.1829
P14	19.75	0.1975
P15	3.87	0.0387
P16	11.19	0.1119
P17	11.70	0.1170
P18	13.07	0.1307
P19	17.80	0.1780
P20	9.12	0.0912
P21	23.72	0.2372
P22	22.51	0.2251
P23	6.46	0.0646
P24	18.97	0.1897
P25	23.39	0.2339
P26	13.79	0.1379
P27	6.46	0.0646
P28	37.11	0.3711
P29	2.28	0.0228

Table S8: Absorbance at 280 nm to determine protein concentration in the final purification supernatant of PtNP trastuzumab samples incubated in human serum using 80 µL of sample (path length = 10 mm).

Sample ID	Absorbance $_{\lambda = 280\ nm}$
P1	0.007
P2	0.003
P3	0.014
P4	0.000
P5	0.007
P6	0.009
P7	0.009
P8	0.007
P9	0.002
P10	0.006
P11	0.004
P12	0.007
P13	0.009
P14	0.014
P15	0.009
P16	0.006
P17	-0.001
P18	0.004
P19	0.007
P20	0.001
P21	0.012
P22	0.005
P23	0.009
P24	0.011
P25	0.003
P26	0.007
P27	0.008
P28	-0.001
P29	0.007

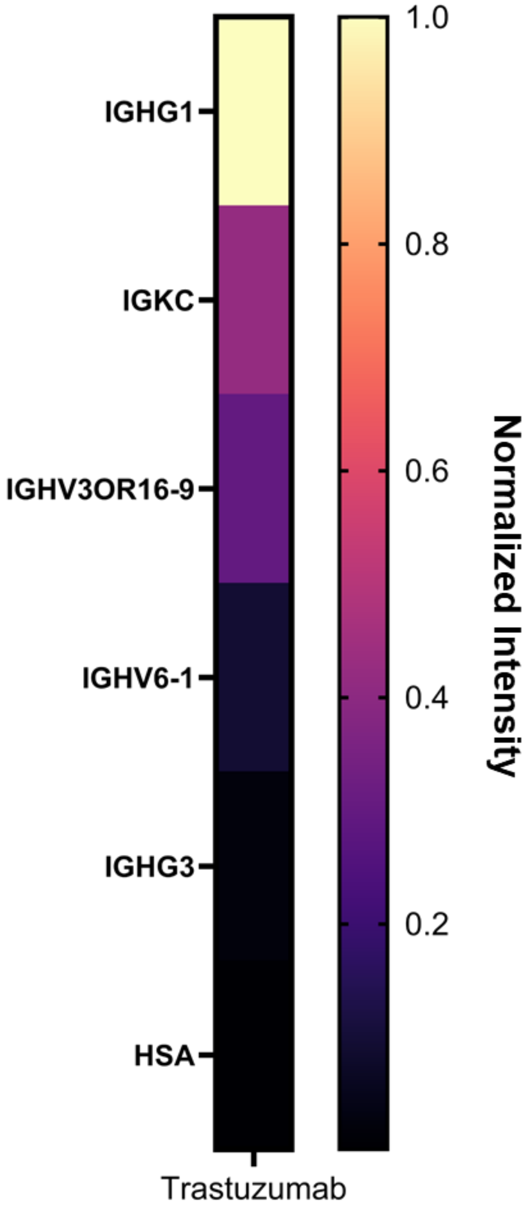

Figure S10: Heatmap illustrating the protein ID of the identified proteins in trastuzumab sample. Intensity values are normalized to intensity of IGHG1.

Table S9: Protein ID of the highest intensity protein identified in RP-nLC-ESI/MS analysis of PtNP trastuzumab samples incubated in human serum patient samples.

**Sample ID**	**Highest Intensity Protein ID**
P1	HSA
P2	HSA
P3	HSA
P4	HSA
P5	HSA
P6	HSA
P7	FN1
P8	APOA1
P9	FN1
P10	FN1
P11	FN1
P12	HSA
P13	FN1
P14	HSA
P15	FN1
P16	HSA
P17	FN1
P18	FN1
P19	HSA
P20	HSA
P21	HSA
P22	HSA
P23	FN1
P24	APOC3
P25	HSA
P26	HSA
P27	FN1
P28	APOA1
P29	IGHG1;IGHG3;IGHG4

Table S10: Number of unique peptides for the top ten highest intensity proteins identified for all 29 human serum patient samples. The number of unique peptides provides an estimate on the confidence of protein identification.

Protein ID	P1	P2	P3	P4	P5	P6	P7	P8	P9	P10	P11	P12	P13	P14	P15	P16	P17	P18	P19	P20	P21	P22	P23	P24	P25	P26	P27	P28	P29
HSA	34	39	34	34	30	35	27	30	21	27	21	37	36	35	23	32	31	23	34	29	36	34	27	17	35	32	27	36	22
APOA1	36	36	35	33	30	32	30	34	26	27	31	36	34	36	29	32	33	34	36	27	34	34	28	32	34	35	33	36	32
APOA2	8	8		7		9		9				8		6					9		7				7	8		8	
APOB	84	92	106				93			101			75	77							68	74				100		85	
APOC3	8	9	8	9	9	7	9		8	9	8	9	9	9	8	8	8	8	7	7	8	8	8	7	8		8	8	7
APOE																16			19	16						14	17		
C3	53	58	61	52	61	41	48	55		56	39	43	52	41	41	56	42	47	51	42	57	56		50	48	51	40		53
C4A;C4B					2			3	30			3					27												2
ECM1																							9						
FGA											15				24			17					29						
FGB									12														17						
FGG																							12						
FN1	71	47	65	33	53	63	73	59	84	64	56	56	72	55	74	45	66	71	63	23	58	48	87	48	39		60	53	44
HP;HPR																										17			
IGHA1;IGHA2																									9				
IGHG1;IGHG3;IGHG4	10	7	4	9	7	7	6	3	9	6	12	11	3	8	7	6	4	3	4	11	9	7	7	7	6	5	9	4	14
IGHV3OR16-9			2	2	1		1	1	1	1	1		2			1	2			1		2					1		1
IGKC	8	9	9	7	8	8	8	8	8	7	9	7	7	9	8	7	7	7	8	7	8	9	6	7	7	8	7	8	7
ITIH4															16									17					
KNG1	11	10	13	10	14	9	8	9	12	10	11	10	12	11	10	11	11	7	10	12	11	11		8	9	12	12	10	11
SERPINA1						17												18						19				20	

Table S11: Protein ID, UniProt ID and protein name for the identified components of the protein corona around PtNP trastuzumab conjugates.

Protein ID	UniProt ID	Protein Name
HSA	P02768	Human serum albumin
APOA1	P02647	Apolipoprotein AI
APOA2	P02652	Apolipoprotein AII
APOB	P04114	Apolipoprotein B
APOC3	P02656	Apolipoprotein CIII
APOE	P02649	Apolipoprotein E
C3	P01024	Complement C3
C4A;C4B	P0C0L4;P0C0L5	Complement C4-A; Complement C4-B
ECM1	Q16610	Extracellular matrix protein 1
FGA	P02671	Fibrinogen alpha chain
FGB	P02675	Fibrinogen beta chain
FGG	P02679	Fibrinogen gamma chain
FN1	P02751	Fibronectin
HP;HPR	P00738;P00739	Haptoglobin; Haptoglobin-related protein
IGHA1;IGHA2	P01876;P01877	Immunoglobulin heavy constant alpha 1; Immunoglobulin heavy constant alpha 2
IGHG1;IGHG3;IGHG4	P01857; P01860; P01861	Immunoglobulin heavy constant gamma 1; Immunoglobulin heavy constant gamma 3; Immunoglobulin heavy constant gamma 4
IGHV3OR16-9	A0A0B4J2B5	Immunoglobulin heavy variable 3/OR16-9
IGKC	P01834	Immunoglobulin kappa constant
ITIH4	Q14624	Inter-alpha-trypsin inhibitor heavy chain H4
KNG1	P01042	Kininogen-1
SERPINA1	P01009	Alpha-1-antitrypsin

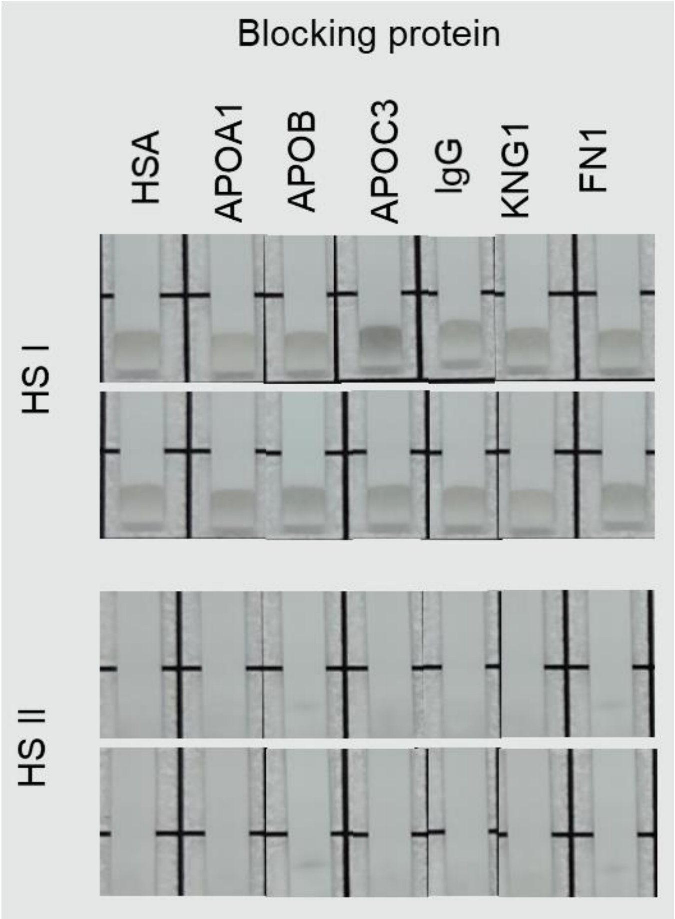

Figure S11: Photographs of LFA strips run in HS I and HS II using isolated human proteins as blocking agents. Samples contain no added antigen (noise), n = 2.

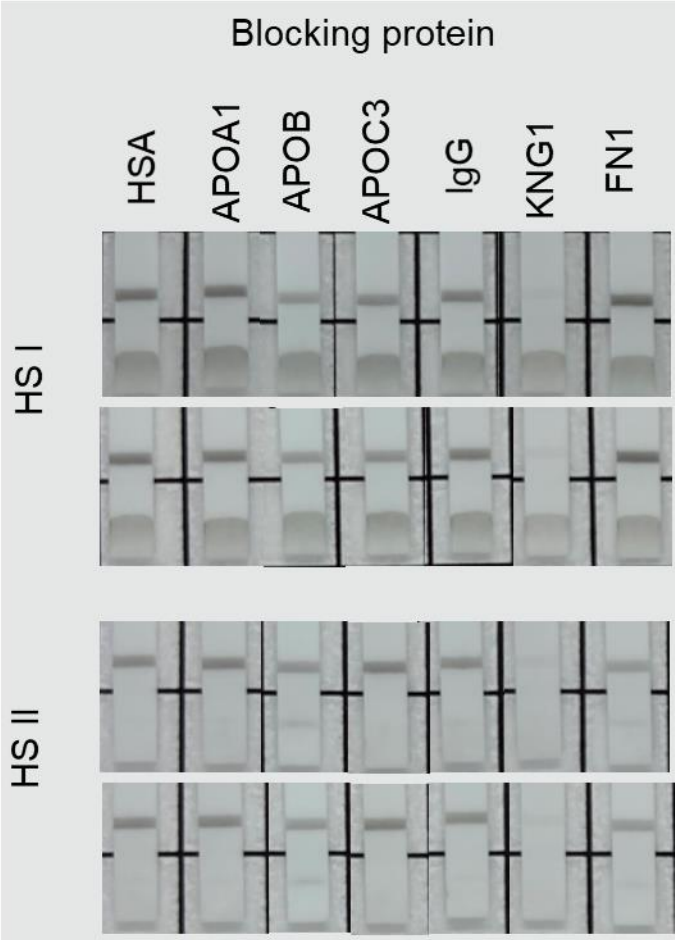

Figure S12: Photographs of LFA strips run in HS I and HS II using isolated human proteins as blocking agents. Samples contain 500 pM of HER2-biotin antigen (signal), n = 2.

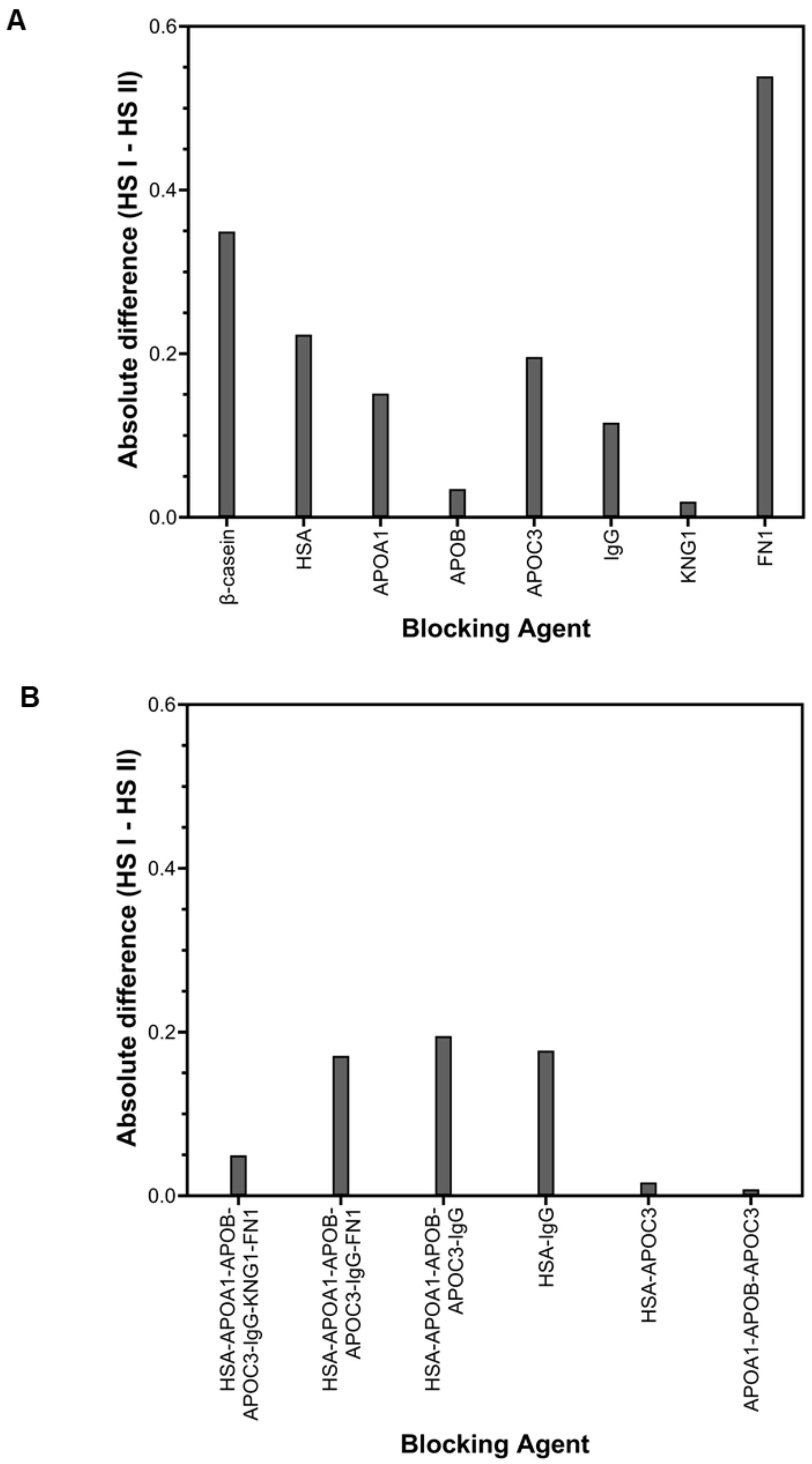

Figure S13: Absolute difference in extracted mean test line intensities for HS I and HS II. A: Absolute difference in observed test line intensity using isolated individual proteins as blocking agents. B: Absolute difference in observed test line intensity using combinations of isolated proteins as blocking agents**.**

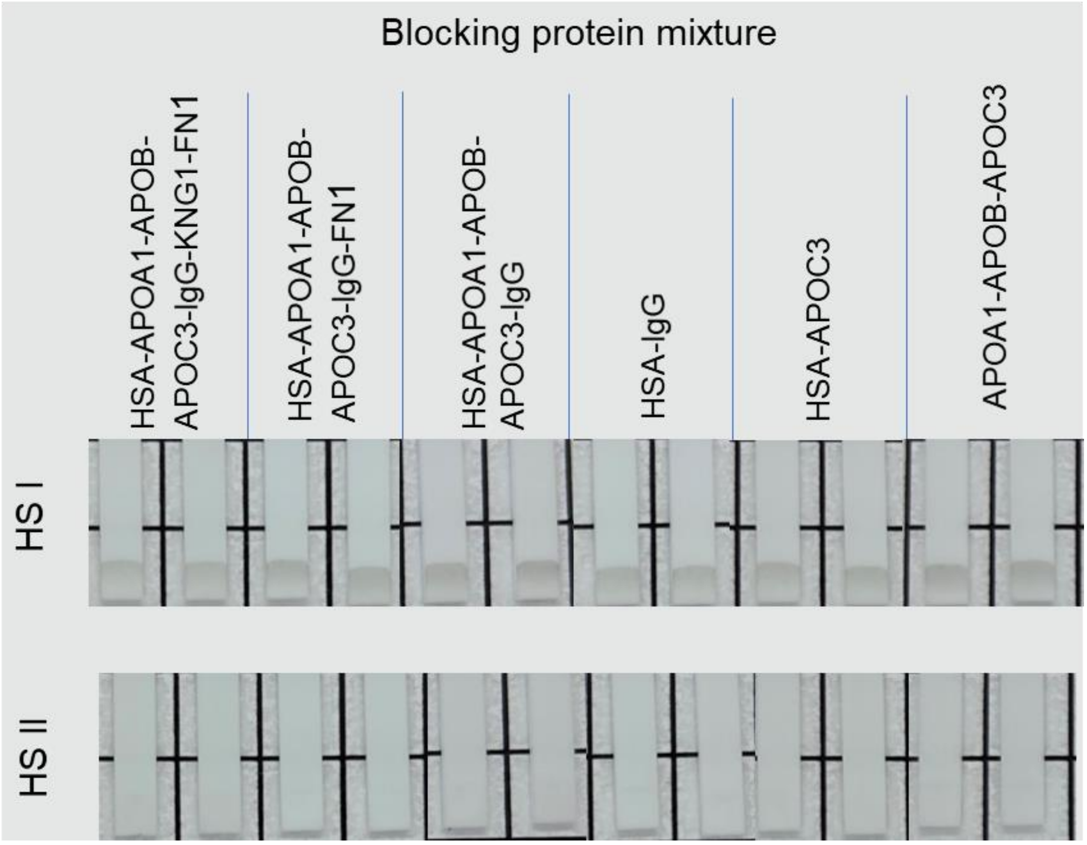

Figure S14: Photographs of LFA strips run in HS I and HS II using combinations of isolated human proteins as blocking agents. Samples contain no added antigen (noise), n = 2.

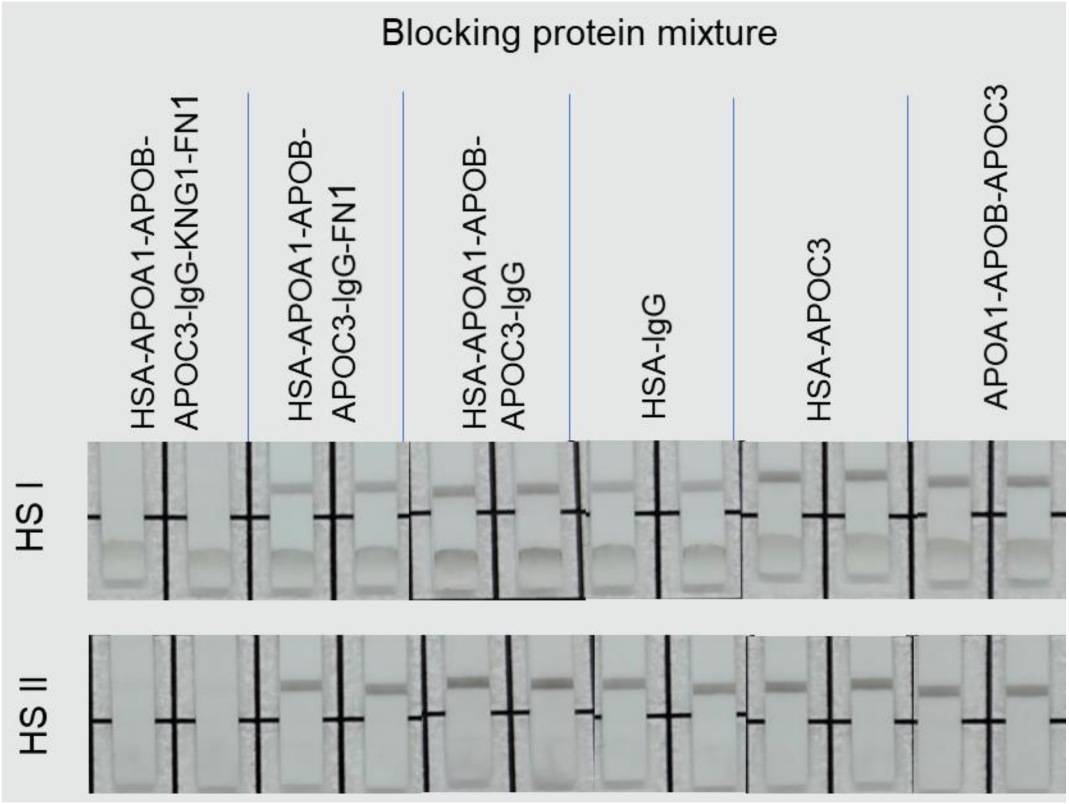

Figure S15: Photographs of LFA strips run in HS I and HS II using combinations of isolated human proteins as blocking agents. Samples contain 500 pM of HER2-biotin antigen (signal), n = 2.

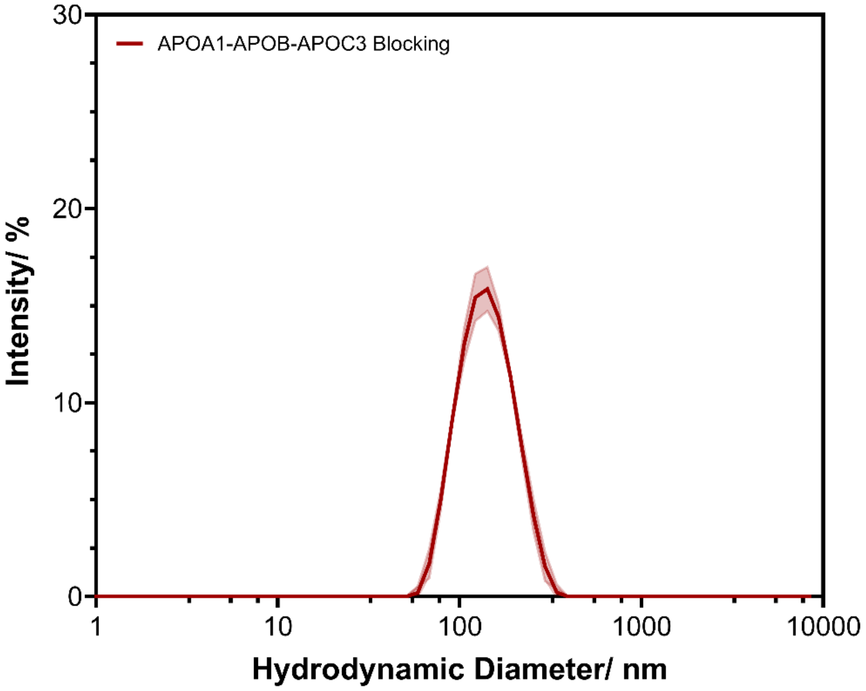

Figure S16: Assessment of hydrodynamic diameter of PtNP conjugate blocked with APOA1-APOB-APOC3. Data plotted as mean ± SD, n = 3.

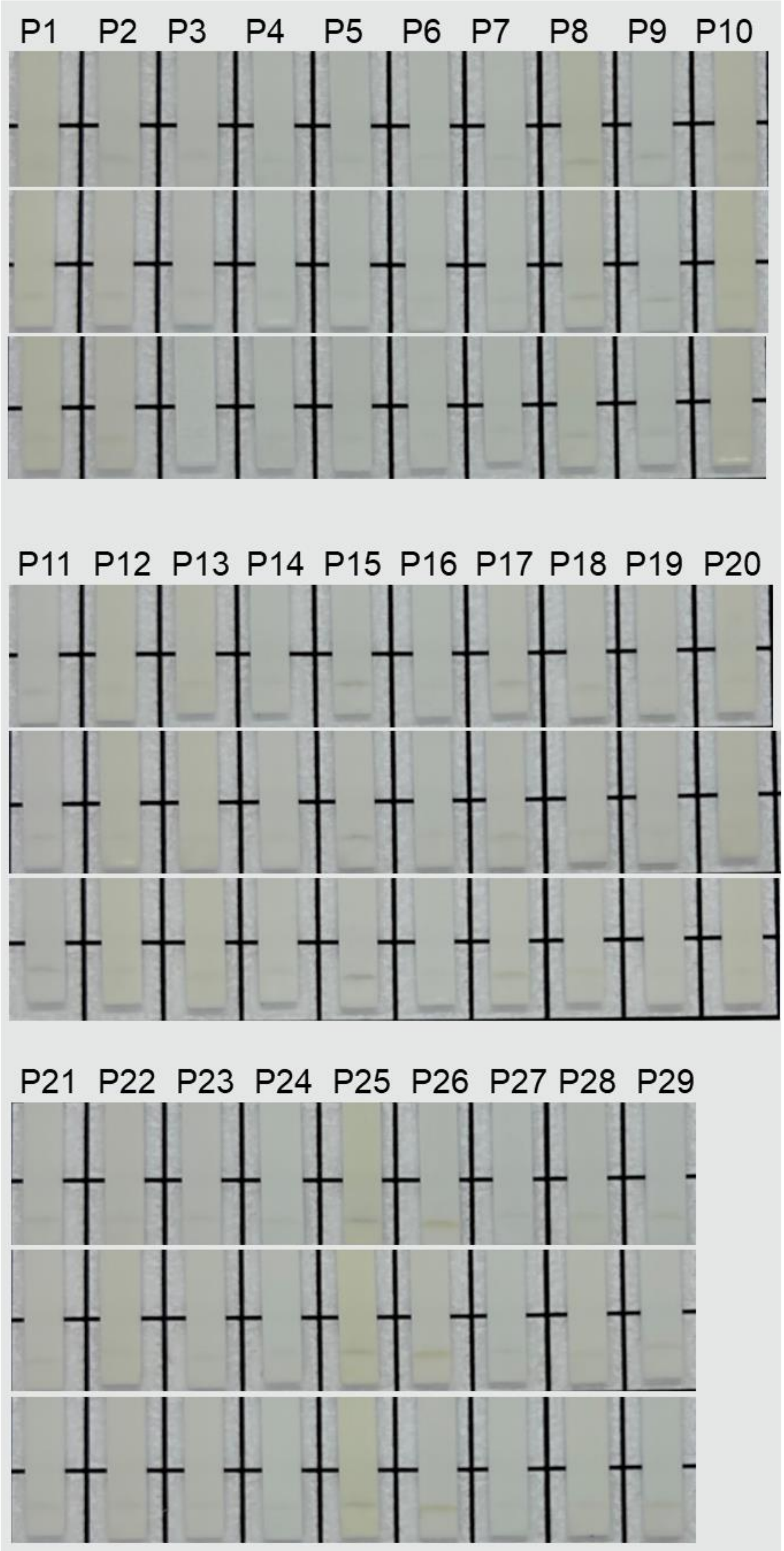

Figure S17: Photographs of LFA test strips using 29 human serum samples with 0 pM HER2-biotin (negative samples) and PtNP detection conjugated with pre-formed APOA1-APOB-APOC3 protein coronas (N = 29, n = 3).

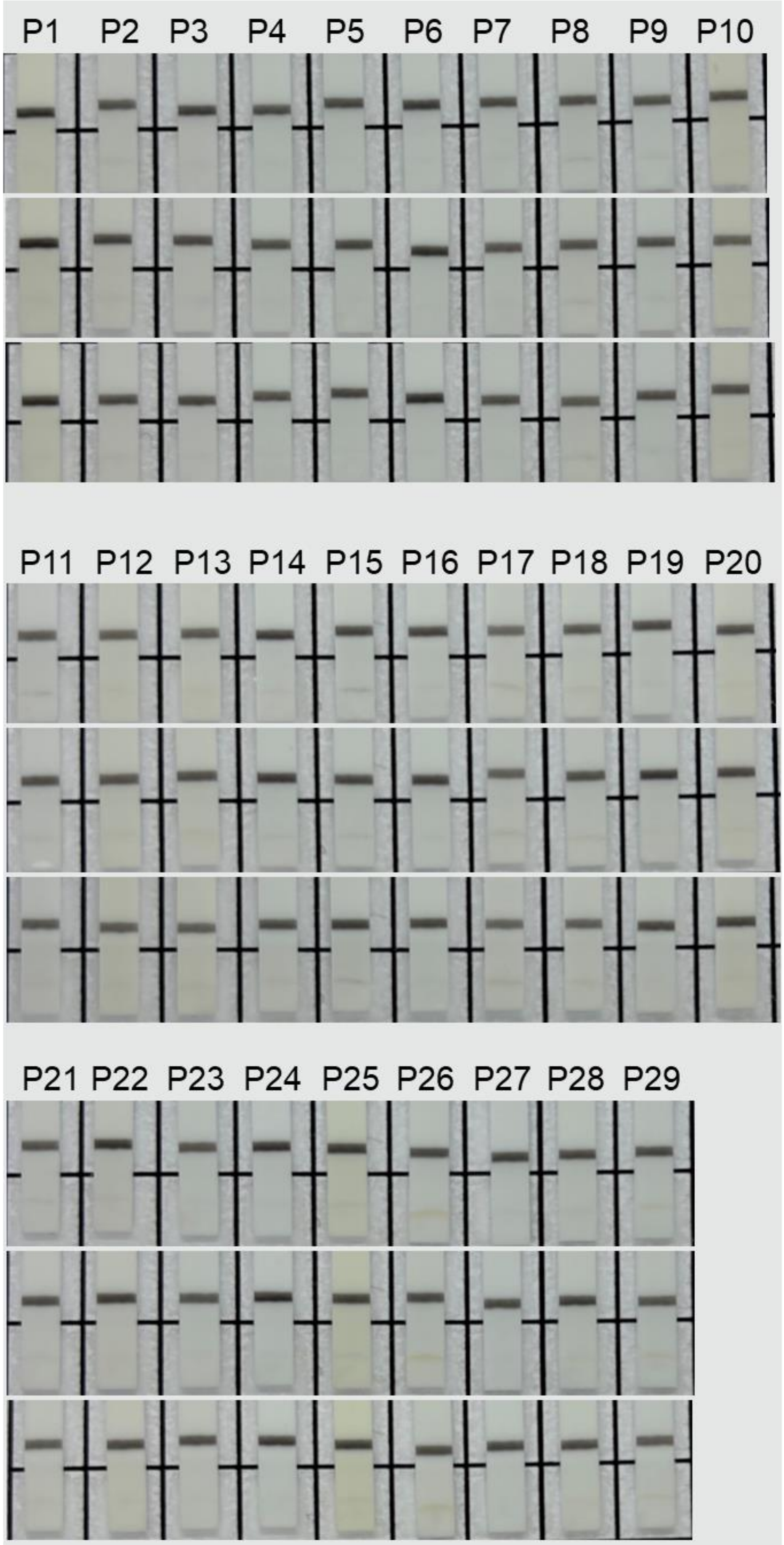

Figure S18: Photographs of LFA test strips using 29 human serum samples with 500 pM HER2-biotin (positive samples) and PtNP detection conjugated with pre-formed APOA1-APOB-APOC3 protein coronas (N = 29, n = 3). The human serum samples used with the LFA were spiked independently to produce the technical replicates.

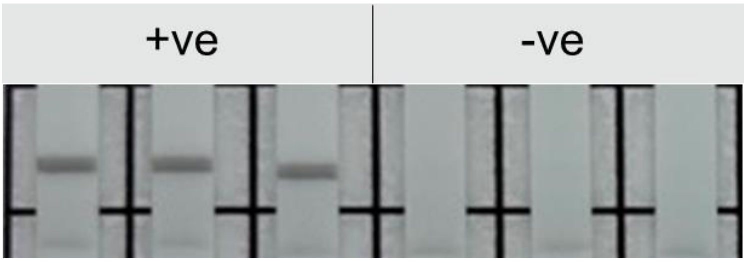

Figure S19: Photographs of LFA test strips using dried and reconstituted pre-formed APOA1-APOB-APOC3 protein coronas with human serum spiked with 500 pM HER2-biotin (+ve) or 0 pM HER2-biotin (-ve), n = 3.

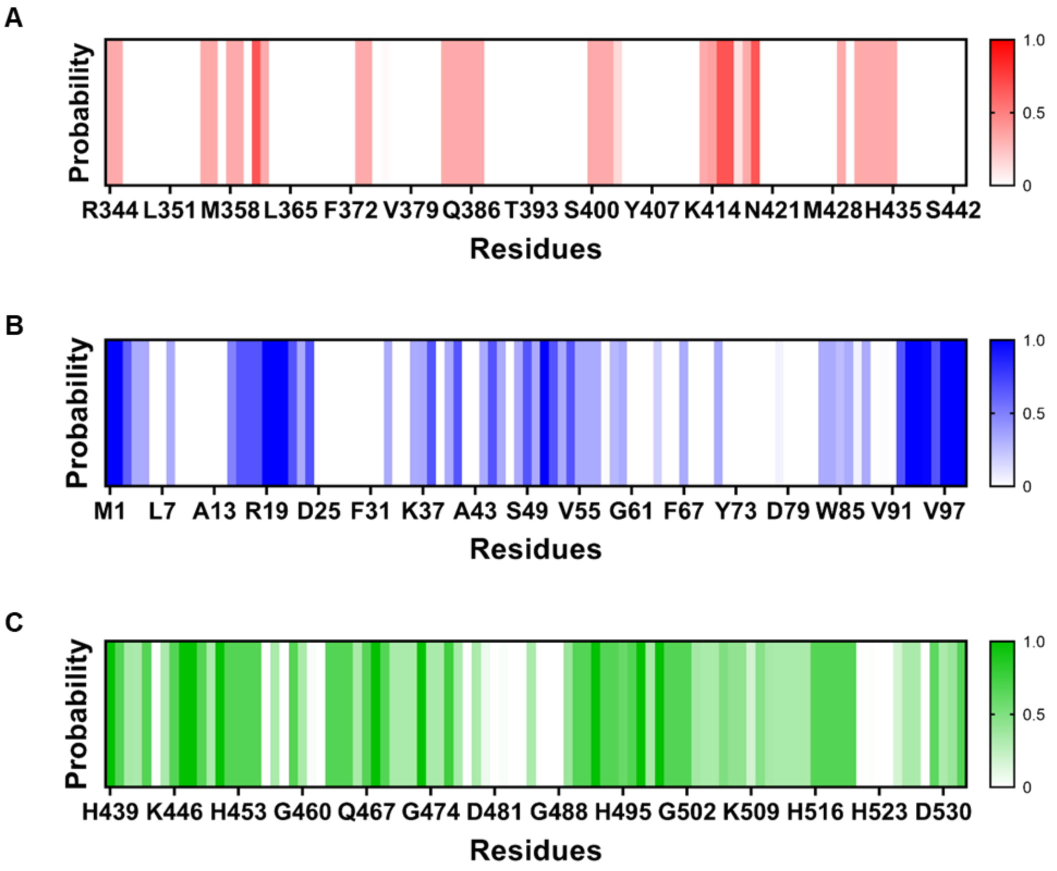

Figure S20: Heatmaps illustrating the surface contact probability of each residue in (A) Fc monomer (red), (B) APOC3 (blue), (C) the surface binding region of KNG-1 (green) to the surface of Pt(111).

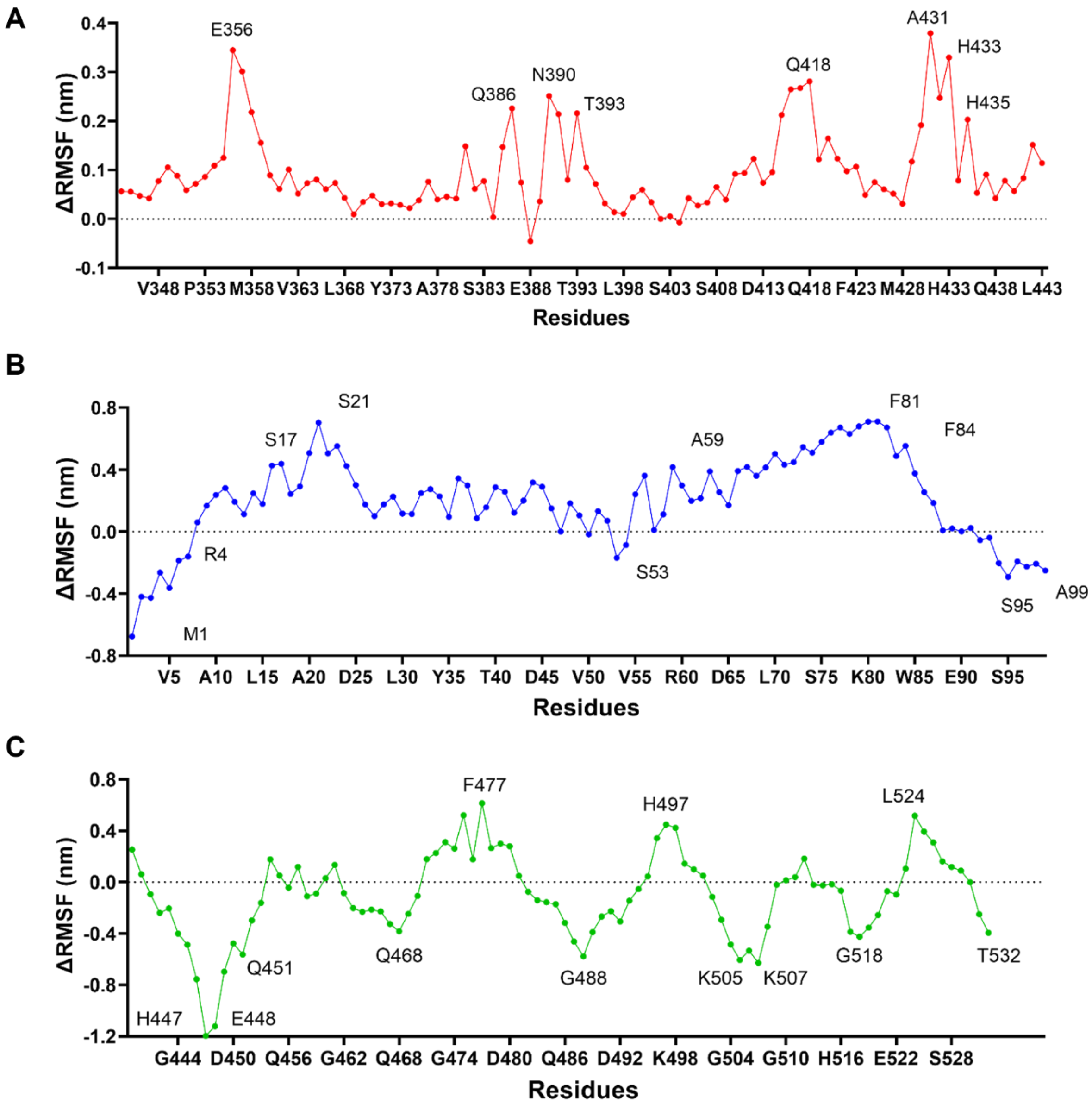

Figure S21: The deviation of the root-mean-square-fluctuation (ΔRMSF) values measured for all residues within the (A) Fc monomer (red), (B) APOC3 (blue), and (C) the surface binding region of KNG1 (green). Values were obtained by subtracting each RMSF value of the individual protein residue in aqueous solution from that on the surface of Pt(111). Residues with ΔRMSF > 0 nm are described as mobile residues. Residues with ΔRMSF < 0 nm are described as rigid residues.

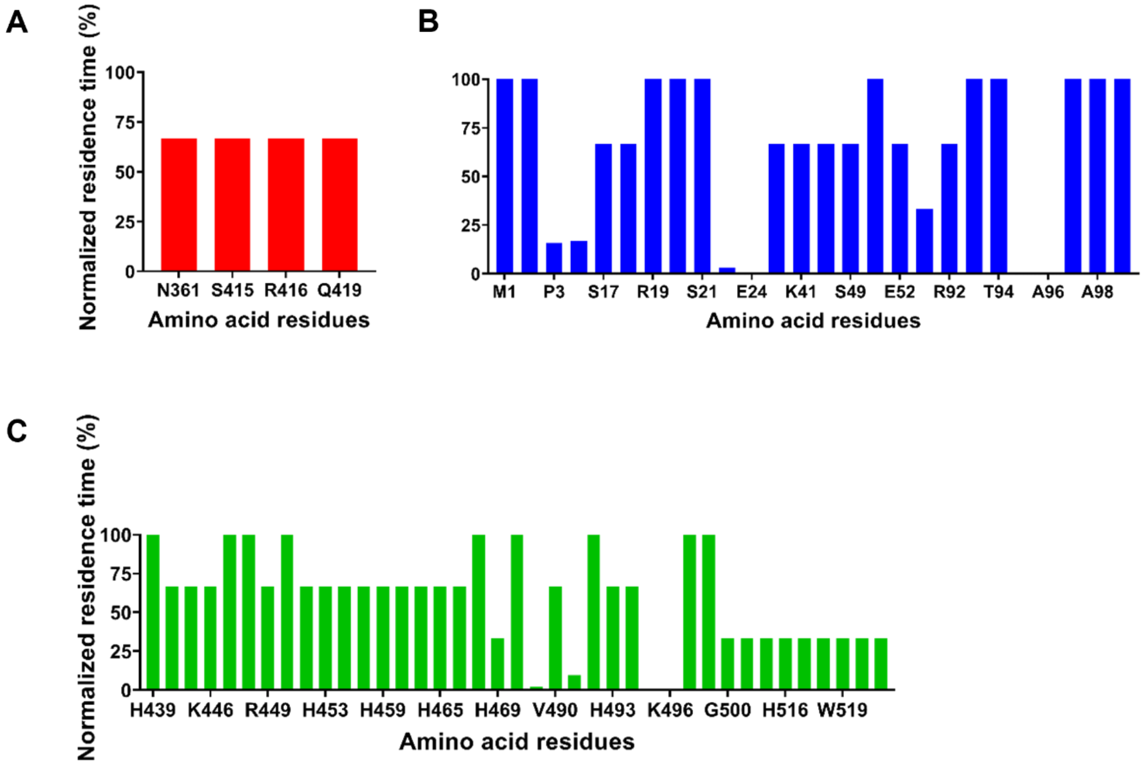

Figure S22: Normalized residence time measured for residues within the (A) Fc monomer (red), (B) APOC3 (blue), and (C) the surface binding region of KNG1 (green) with a contact probability of above 0.5.

Supporting Information Experimental Section:

Transmission electron microscopy (TEM)

Sample preparation for TEM characterization was performed by diluting bare PtNPs in UPDW (100 pM). To an ultrathin carbon support film on lacey carbon grids (Agar Scientific), 2 µL of the sample was drop-casted and left to air-dry for 30 min.

TEM imaging was performed using a JEOL JEM-2100, operating at 200 kV. Images were processed in Fiji to individually adjust the brightness.

Dynamic light scattering (DLS)

Dynamic light scattering measurements were performed on a Zetasizer Nano ZS (Malvern Instruments, Ltd.) equipped with a 633 nm He-Ne laser. Measurement parameters were set using the Zetasizer Nano software v8.02. 100 µL of sample volumes were used for all experiments.

UV-Vis spectroscopy

The UV-vis spectra of protein samples were measured using 80 µL of the sample (cuvette mode) or 2 µL of the sample (droplet mode). The absorbance at 280 nm was used to probe protein concentration (NanoDrop 2000c, Thermo Scientific).

MicroBCA assay

MicroBCA assays were used to assess the concentration of protein in the final supernatant washing of samples prior to RP-nLC-ESI/MS analysis. The concentration of protein in the supernatant represents the concentration of serum proteins that are not bound to the PtNPs. The microBCA assay was performed according to the manufacturer's microplate protocol (catalog number: 23235). A bovine serum albumin (BSA) standard curve was used to extract protein concentrations.

Preparation of protein samples for RP-nLC/ESI-MS analysis

Protein samples (trastuzumab, HS I and HS II) were diluted to ca. 1 mg mL^{-1} in DPBS, with a sample volume of 10 µL. Human serum samples were assumed to have a protein concentration of

ca. 0 mg mL^{-1}. The samples were dried using a vacuum concentrator (Eppendorf) with the temperature set to 45 °C to remove all solvent.

Samples were resuspended in 10 µL of 8 M urea (Sigma) in 100 mM ammonium bicarbonate buffer (Sigma) before incubation in an ultrasonic water bath for 30 min. The samples were removed before the addition of 0.52 µL of TCEP solution (20 mM TCEP in 100 mM ammonium bicarbonate buffer, Sigma) and incubation at 56 °C for 1 h. The samples were cooled to RT before the addition of 0.58 µL of iodoacetamide (84 mM iodoacetamide in 100 mM ammonium bicarbonate buffer, Sigma) and incubated in the dark at RT for 1 h. The final concentration of urea was diluted to 1 M by the addition of 79 µL of 100 mM ammonium bicarbonate buffer. The sample was digested by adding 1 µL of sequencing-grade trypsin (40 ng µL^{-1} in 50 mM acetic acid, Promega) and allowed to incubate overnight at 37 °C under shaking (300 rpm). The digestion was stopped by adding 5 µL of neat formic acid (Fisher).

The samples were desalted using C18 ZipTip pipette tips (Sigma). Briefly, a p200 pipette was used to pick up the ZipTip, and the pipette volume was set to 110 µL. The ZipTip was washed 5x with 80 v/v% acetonitrile (ACN) (LC/MS grade, Sigma) using a total volume of 500 µL. The ZipTip was equilibrated 5x with H_2O (LC/MS grade, Sigma) + 1 v/v% formic acid (FA) (Fisher) using a total volume of 500 µL. The digested sample was loaded onto the ZipTip by aspirating the sample over the C18 resin 3x. The loaded ZipTip was washed 2x with H_2O (LC/MS grade, Sigma) + 1 v/v% FA (Fisher), where the washed sample was reserved in a 1.5 mL Protein LoBind Eppendorf. The sample was eluted from the C18 resin 2x by aspiration of 80 v/v% ACN (LC/MS grade, Sigma) + 1 v/v% FA (Fisher). The sample was dried using a vacuum concentrator (Eppendorf) with the temperature set to 45 °C to remove all solvent. The dry sample was stored at -20 °C before analysis.

Preparation of PtNP trastuzumab-human serum samples for RP-nLC/ESI-MS analysis

PtNP were functionalized with trastuzumab prior to sample digestion. To a 2 mL Protein LoBind sample tube (Eppendorf), 600 µL of PtNC (300 pM) was added, followed by 60 µL of conjugation buffer (100 mM carbonate buffer, pH 9) and 10.80 µL of trastuzumab (1 mg mL^{-1}, Biosynth). The mixture was briefly vortexed and incubated for 3 h at RT under shaking (700 rpm). Excess reagents were removed through 3x washing steps, whereby conjugates were centrifuged at 5000 rcf for 5 min to form a pellet, the supernatant removed, and conjugates resuspended in 1000 µL of DPBS. After the final wash step, the conjugate was resuspended to a total volume of 100 µL (1800 pM) and stored at 4 °C.

The PtNP trastuzumab conjugate was then incubated in 100 µL of pooled human serum for 15 min under shaking (700 rpm). Excess reagents were removed through 4x washing steps, whereby conjugates were centrifuged at 5000 rcf for 5 min to form a pellet, the supernatant removed, and conjugates resuspended in 1000 µL of DPBS. After the final wash step, the supernatant was removed, leaving a final conjugate volume of 10 µL. The conjugates were dried using a vacuum concentrator (Eppendorf) with the temperature set to 45 °C to remove all solvent.

Samples were resuspended in 10 µL of 8 M urea (Sigma) in 100 mM ammonium bicarbonate buffer (Sigma) before incubation in an ultrasonic water bath for 30 min. The samples were removed before the addition of 0.52 µL of TCEP solution (20 mM TCEP in 100 mM ammonium bicarbonate buffer, Sigma) and incubation at 56 °C for 1 h. The samples were cooled to RT before the addition of 0.58 µL of iodoacetamide (84 mM iodoacetamide in 100 mM ammonium bicarbonate buffer, Sigma) and incubated in the dark at RT for 1 h. The final concentration of urea was diluted to 1 M by the addition of 79 µL of 100 mM ammonium bicarbonate buffer. The sample was digested by adding 1 µL of sequencing-grade trypsin (40 ng μL^{-1} in 50 mM acetic acid, Promega) and allowed to incubate overnight at 37 °C under shaking (300 rpm). The digestion was stopped by adding 5 µL of neat formic acid (Fisher).

The samples were desalted using C18 ZipTip pipette tips (Sigma). Briefly, a p200 pipette was used to pick up the ZipTip, and the pipette volume was set to 110 µL. The ZipTip was washed 5x with 80 v/v% acetonitrile (ACN, LC/MS grade, Sigma) using a total volume of 500 µL. The ZipTip was equilibrated 5x with H_2O (LC/MS grade, Sigma) + 1 v/v% formic acid (FA, Fisher UK) using a total volume of 500 µL. The digested sample was loaded onto the ZipTip by aspirating the sample over the C18 resin 3x. The loaded ZipTip was washed 2x with H_2O (LC/MS grade, Sigma) + 1 v/v% formic acid (Fisher), where the washed sample was reserved in a 1.5 mL Protein LoBind Eppendorf. The sample was eluted from the C18 resin 2x by aspiration of 80 v/v% ACN (LC/MS grade, Sigma) + 1 v/v% FA (Fisher). The sample was dried using a vacuum concentrator (Eppendorf) with the temperature set to 45 °C to remove all solvent. The dry sample was stored at -20 °C before analysis.

Reversed-Phase nano Liquid Chromatography coupled with Electrospray Ionization Mass Spectrometry (RP-nLC/ESI-MS) conditions

Digested protein samples were rehydrated in 20 µL of mobile phase A (0.1 v/v% formic acid (FA) in water (LC/MS grade)).

The nLC Dionex UltiMate 3000 RSLC system was operated at a flow rate of 300 nL min^{-1} using the following gradient:

Time/ min	Gradient of mobile phase B
0-4	0-5%
4-35	5-35%
35-38	35-99%
38-43	99%
43-45	5%
45-50 (column re-equilibration)	5%

Mobile phase solvents were purchased from Sigma and were LC/MS Grade. Mobile phase A was 0.1 v/v% formic acid (FA) in water. Mobile phase B was 80.0:20.0 0.1 v/v% acetonitrile (ACN)/ 0.1 v/v% FA.

Peptide separation was performed on an Acclaim PepMap C18 column with 3 µm particle size, 75 µm x 15 cm, 100 Å (Thermo Fisher Scientific, catalog number: 164568). A trapping column (PepMap 100 C18 3 µm x 75 µm x 2 cm (particle size x length x diameter), Thermo Fisher Scientific, catalog number: 164946) was used in-line with the LC prior to separation with the analytical column.

The Orbitrap Eclipse mass spectrometer (Thermo Fischer Scientific) was operated under peptide mode. The global settings were as follows: NanoFlex ion source with 50 µm inner diameter stainless steel emitter, positive voltage of 2200 V, and an Ion Transfer Tube Temp of 320 °C. Ions for the MS scans were detected in the Orbitrap with a resolution of 120,000. The mass range was normal, quadrupole isolation was checked, and the scan range was set to 375-1500 m/z. The source RF amplitude was set to 45 %, and the AGC target was 4.0 x 10^5. One microscan per scan was taken.

The following filters were utilized: MIPS, Charge State, and Dynamic Exclusion. The MIPS filter was set to peptide. The charge state filter was set to include charge states 2-7. A dynamic exclusion filter was configured to exclude ions after 2 times for a duration of 20 s with a mass tolerance of ± 10 ppm. Isotopes were excluded. Following the dynamic exclusion filter, the method branched into two ddMS2 legs with respective charge state filters. Electron-transfer high-energy collision dissociation (EThcD) was used for fragmentation. EThcD reaction kinetics were determined with prior calibration using Pierce FlexMix with supplemental higher energy collision dissociation set to 15%. Precursor and fragment detection were performed using an Orbitrap at a resolution MS1 = 120,000 and MS2 = 30,000.

The AGC target for MS1 was set to standard, and the injection time was set to auto, which involves the system setting the two parameters to maximize sensitivity whilst maintaining cycle time.

Production of PtNP trastuzumab conjugates with engineered protein corona

The ratio of isolated human protein to PtNP was calculated using the trastuzumab: PtNP ratio of 400:1, and scaled according to isolated protein molecular weight.

Table M1: Isolated human proteins used as PtNP blocking agents to pre-form an engineered protein corona. The protein to PtNP ratio is adjusted according to protein molecular weight.

Protein and Catalog Number	Molecular Weight/ kDa	Protein: PtNP ratio
HSA (Sigma)<br>A9511	66.5	900:1
APOA1 (Sigma)<br>SRP4693	28	2150:1
APOB (Sigma)<br>ALP30	550	110:1
APOC3 (Merck)<br>178461	8.8	6900:1
IgG (Merck)<br>I4506	150	400:1
KNG1 (Creative Biomart)<br>KNG1-1844H	45	1350:1
FN1 (Sigma)<br>F0895	260	230:1

Table M2: Combinations of isolated human proteins used as PtNP blocking agents to pre-form an engineered protein corona. The protein to PtNP ratio is adjusted according to protein molecular weight.

Protein Mixture	Protein: PtNP ratio
HSA-APOA1-APOB-APOC3-IgG-KNG1-FN1	900:1 – 2150:1 – 110:1 – 6900:1 – 400:1 – 1350:1 – 230:1
HSA-APOA1-APOB-APOC3-IgG -FN1	900:1 – 2150:1 – 110:1 – 6900:1 – 400:1 – 230:1
HSA-APOA1-APOB-APOC3-IgG	900:1 – 2150:1 – 110:1 – 6900:1 – 400:1
HSA-IgG	900:1 – 400:1
HSA-APOC3	900:1 – 6900:1
APOA1-APOB-APOC3	2150:1 – 110:1 – 6900:1

Molecular Dynamics Simulations Supporting Information

Construction of protein models

The structures for both the Fc monomer (PDB ID: 1H3Y) and the APOC3 (PDB ID: 2JQ3) were obtained from the Protein Data Bank.[1,2] For the Fc monomer, residues 344 to 444, representing the CH3 component, were selected, while the missing loop in the APOC3 was constructed using MODELLER 10.6.[3] The structure of the KNG1 protein was sourced from AlphaFold (AF-P01042-F1), and residues 439 to 532, which have been experimentally identified as the surface binding region, were chosen for modelling.[4,5]

Molecular dynamics simulations for proteins in aqueous solution

Classical molecular dynamics (MD) simulations were performed using the GROMACS 2021.4 suite in conjunction with the CHARMM36m force field.[6–8] TIP3P water molecules were used to solvate each system in a cubic box with a minimum distance of 2.0 nm between the edge of the box and any protein atoms. Sodium chloride ions at a concentration of 0.14 M and counter ions were added to salt and neutralize the system. Energy minimization was performed using the steepest-descent algorithm for 10,000 steps. Throughout all simulations, periodic boundary conditions were applied with bond lengths constrained using the LINCS algorithm, enabling an integration timestep of 2 fs.[9] Subsequent simulations under canonical (NVT) and isothermal-isobaric (NPT) ensembles were conducted, where all heavy atoms within the proteins were restrained for 500 ps using the modified Berendsen thermostat and barostat, consecutively.[10,11] Long-range electrostatic interactions were calculated using the particle-mesh Ewald (PME) scheme with a grid spacing of 0.12 nm, while both Coulomb and van der Waals interactions were truncated at a distance of 1.2 nm.[12] Final MD simulations with a random seed were performed for 200 ns. During these production runs, the temperature and pressure of each system were regulated using the v-rescale thermostat and Parinello-Rahman barostat, respectively.[11,13–15]

Molecular dynamics simulations for proteins on the Pt(111) surface

Similarly, MD simulations were performed using GROMACS 2021.4 to model the protein-surface complexes, which included a six-layer Pt(111) slab, a protein molecule, explicit aqueous solvent, and salt ions.[6,8] The fundamental simulation parameters, such as the algorithms used and the cut-off values for electrostatic and van der Waals interactions, were adapted from the previous section. The INTERFACE force field was used to compute the intermolecular interactions between the protein and Pt(111).[16] The unit cell of lateral xy dimensions was approximately 12×12 nm. Periodic boundary

conditions were implemented with a vacuum spacer of 10 nm along the perpendicular z-direction from the Pt(111) surface to omit any interlayer interactions. For each system, three different initial conformations obtained from the simulations in aqueous solution were placed approximately 0.9 nm above the Pt(111) surface to minimize potential bias from the starting orientation. The TIP3P water model was used to solvate the system, and NaCl was added at a concentration of 0.14 M along with counter ions to ensure charge neutrality. Energy minimization was then conducted, employing the steepest-descent algorithm for 10,000 steps. This was then followed by preliminary equilibration with an integration timestep of 1 fs under NVT and NPT ensembles, in which all heavy atoms within the proteins were restrained for 500 ps using the modified Berendsen thermostat and barostat, consecutively.[10,11] Subsequent equilibration steps were carried out for 500 ps under the NPT ensemble with an integration timestep of 2 fs, where the restraint force constant was reduced to 500 kJ mol^{-1} nm^{-2} and then to 250 kJ mol^{-1} nm^{-2}, respectively. Finally, MD simulations were performed for 500 ns using the Nose-Hoover thermostat and Parinello-Rahman barostat to collect the data for analyses.[13–15,17,18]

References:

(1) Gangabadage, C. S.; Zdunek, J.; Tessari, M.; Nilsson, S.; Olivecrona, G.; Wijmenga, S. S. Structure and Dynamics of Human Apolipoprotein CIII. *Journal of Biological Chemistry* **2008**, *283* (25), 17416–17427. https://doi.org/10.1074/jbc.M800756200.

(2) Krapp, S.; Mimura, Y.; Jefferis, R.; Huber, R.; Sondermann, P. Structural Analysis of Human IgG-Fc Glycoforms Reveals a Correlation Between Glycosylation and Structural Integrity. *J. Mol. Biol.* **2003**, *325* (5), 979–989. https://doi.org/10.1016/S0022-2836(02)01250-0.

(3) Šali, A.; Blundell, T. L. Comparative Protein Modelling by Satisfaction of Spatial Restraints. *J. Mol. Biol.* **1993**, *234* (3), 779–815. https://doi.org/10.1006/jmbi.1993.1626.

(4) Jumper, J.; Evans, R.; Pritzel, A.; Green, T.; Figurnov, M.; Ronneberger, O.; Tunyasuvunakool, K.; Bates, R.; Žídek, A.; Potapenko, A.; Bridgland, A.; Meyer, C.; Kohl, S. A. A.; Ballard, A. J.; Cowie, A.; Romera-Paredes, B.; Nikolov, S.; Jain, R.; Adler, J.; Back, T.; Petersen, S.; Reiman, D.; Clancy, E.; Zielinski, M.; Steinegger, M.; Pacholska, M.; Berghammer, T.; Bodenstein, S.; Silver, D.; Vinyals, O.; Senior, A. W.; Kavukcuoglu, K.; Kohli, P.; Hassabis, D. Highly Accurate Protein Structure Prediction with AlphaFold. *Nature* **2021**, *596* (7873), 583–589. https://doi.org/10.1038/s41586-021-03819-2.

(5) Schousboe, I.; Nystrøm, B. High Molecular Weight Kininogen Binds to Laminin – Characterization and Kinetic Analysis. *FEBS J.* **2009**, *276* (18), 5228–5238. https://doi.org/10.1111/j.1742-4658.2009.07218.x.

(6) Abraham, M. J.; Murtola, T.; Schulz, R.; Páll, S.; Smith, J. C.; Hess, B.; Lindahl, E. GROMACS: High Performance Molecular Simulations through Multi-Level Parallelism from Laptops to Supercomputers. *SoftwareX* **2015**, *1–2*, 19–25. https://doi.org/10.1016/j.softx.2015.06.001.

(7) Bjelkmar, P.; Larsson, P.; Cuendet, M. A.; Hess, B.; Lindahl, E. Implementation of the CHARMM Force Field in GROMACS: Analysis of Protein Stability Effects from Correction Maps, Virtual Interaction Sites, and Water Models. *J. Chem. Theory Comput.* **2010**, *6* (2), 459–466. https://doi.org/10.1021/ct900549r.

(8) Hess, B.; Kutzner, C.; van der Spoel, D.; Lindahl, E. GROMACS 4: Algorithms for Highly Efficient, Load-Balanced, and Scalable Molecular Simulation. *J. Chem. Theory Comput.* **2008**, *4* (3), 435–447. https://doi.org/10.1021/ct700301q.

(9) Hess, B.; Bekker, H.; Berendsen, H. J. C.; Fraaije, J. G. E. M. LINCS: A Linear Constraint Solver for Molecular Simulations. *J. Comput. Chem.* **1997**, *18* (12), 1463–1472. https://doi.org/10.1002/(SICI)1096-987X(199709)18:12<1463::AID-JCC4>3.0.CO;2-H.

(10) Berendsen, H. J. C.; Postma, J. P. M.; van Gunsteren, W. F.; DiNola, A.; Haak, J. R. Molecular Dynamics with Coupling to an External Bath. *J. Chem. Phys.* **1984**, *81* (8), 3684–3690. https://doi.org/10.1063/1.448118.

(11) Bussi, G.; Donadio, D.; Parrinello, M. Canonical Sampling through Velocity Rescaling. *J. Chem. Phys.* **2007**, *126* (1). https://doi.org/10.1063/1.2408420.

(12) Essmann, U.; Perera, L.; Berkowitz, M. L.; Darden, T.; Lee, H.; Pedersen, L. G. A Smooth Particle Mesh Ewald Method. *J. Chem. Phys.* **1995**, *103* (19), 8577–8593. https://doi.org/10.1063/1.470117.

(13) Parrinello, M.; Rahman, A. Crystal Structure and Pair Potentials: A Molecular-Dynamics Study. *Phys. Rev. Lett.* **1980**, *45* (14), 1196–1199. https://doi.org/10.1103/PhysRevLett.45.1196.

(14) Parrinello, M.; Rahman, A. Polymorphic Transitions in Single Crystals: A New Molecular Dynamics Method. *J. Appl. Phys.* **1981**, *52* (12), 7182–7190. https://doi.org/10.1063/1.328693.

(15) Parrinello, M.; Rahman, A. Strain Fluctuations and Elastic Constants. *J. Chem. Phys.* **1982**, *76* (5), 2662–2666. https://doi.org/10.1063/1.443248.

(16) Heinz, H.; Lin, T.-J.; Kishore Mishra, R.; Emami, F. S. Thermodynamically Consistent Force Fields for the Assembly of Inorganic, Organic, and Biological Nanostructures: The INTERFACE Force Field. *Langmuir* **2013**, *29* (6), 1754–1765. https://doi.org/10.1021/la3038846.

(17) Hoover, W. G. Canonical Dynamics: Equilibrium Phase-Space Distributions. *Phys. Rev. A (Coll Park).* **1985**, *31* (3), 1695–1697. https://doi.org/10.1103/PhysRevA.31.1695.

(18) Nosé, S. A Molecular Dynamics Method for Simulations in the Canonical Ensemble. *Mol. Phys.* **1984**, *52* (2), 255–268. https://doi.org/10.1080/00268978400101201.
